# mRNA nanodelivery systems: targeting strategies and administration routes

**DOI:** 10.1186/s40824-023-00425-3

**Published:** 2023-09-22

**Authors:** Mujie Yuan, Zeyu Han, Yan Liang, Yong Sun, Bin He, Wantao Chen, Fan Li

**Affiliations:** 1 Department of Oral Implantology, The Affiliated Hospital of Qingdao University, Qingdao, 266000 China; 2 Department of Pharmaceutics, School of Pharmacy, Qingdao University, Qingdao, 266073 China; 3 National Engineering Research Center for Biomaterials, Sichuan University, Chengdu, 610064 China; 4 Department of Oral and Maxillofacial-Head & Neck Oncology, Shanghai Ninth People’s Hospital, Shanghai Jiao Tong University School of Medicine, Shanghai, 200011 China

**Keywords:** mRNA, Nanodelivery systems, Targeting, Administration routes

## Abstract

With the great success of coronavirus disease (COVID-19) messenger ribonucleic acid (mRNA) vaccines, mRNA therapeutics have gained significant momentum for the prevention and treatment of various refractory diseases. To function efficiently in vivo and overcome clinical limitations, mRNA demands safe and stable vectors and a reasonable administration route, bypassing multiple biological barriers and achieving organ-specific targeted delivery of mRNA. Nanoparticle (NP)-based delivery systems representing leading vector approaches ensure the successful intracellular delivery of mRNA to the target organ. In this review, chemical modifications of mRNA and various types of advanced mRNA NPs, including lipid NPs and polymers are summarized. The importance of passive targeting, especially endogenous targeting, and active targeting in mRNA nano-delivery is emphasized, and different cellular endocytic mechanisms are discussed. Most importantly, based on the above content and the physiological structure characteristics of various organs in vivo, the design strategies of mRNA NPs targeting different organs and cells are classified and discussed. Furthermore, the influence of administration routes on targeting design is highlighted. Finally, an outlook on the remaining challenges and future development toward mRNA targeted therapies and precision medicine is provided.

**Graphical Abstract:**

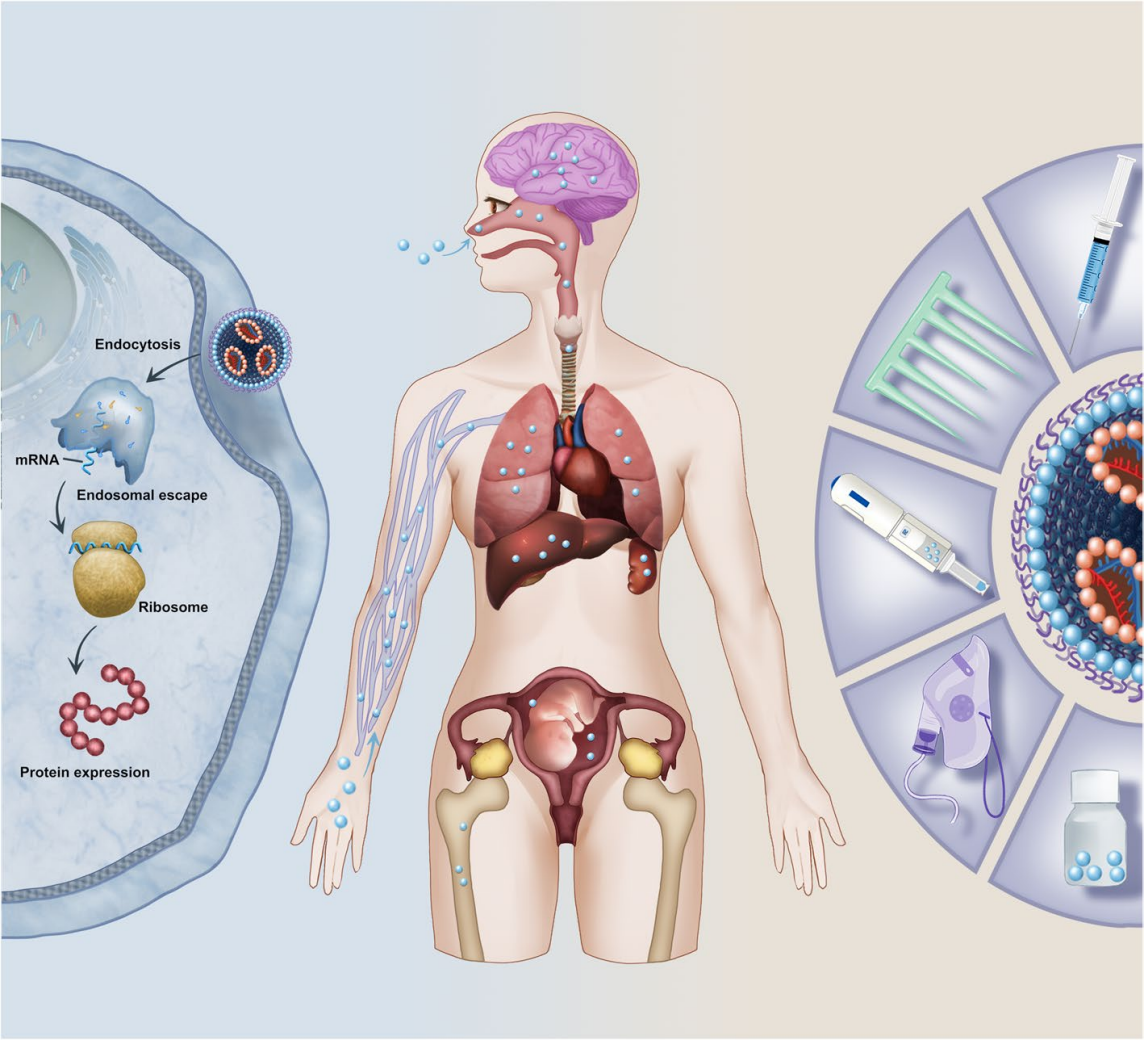

## Introduction

**Fig. 1:**
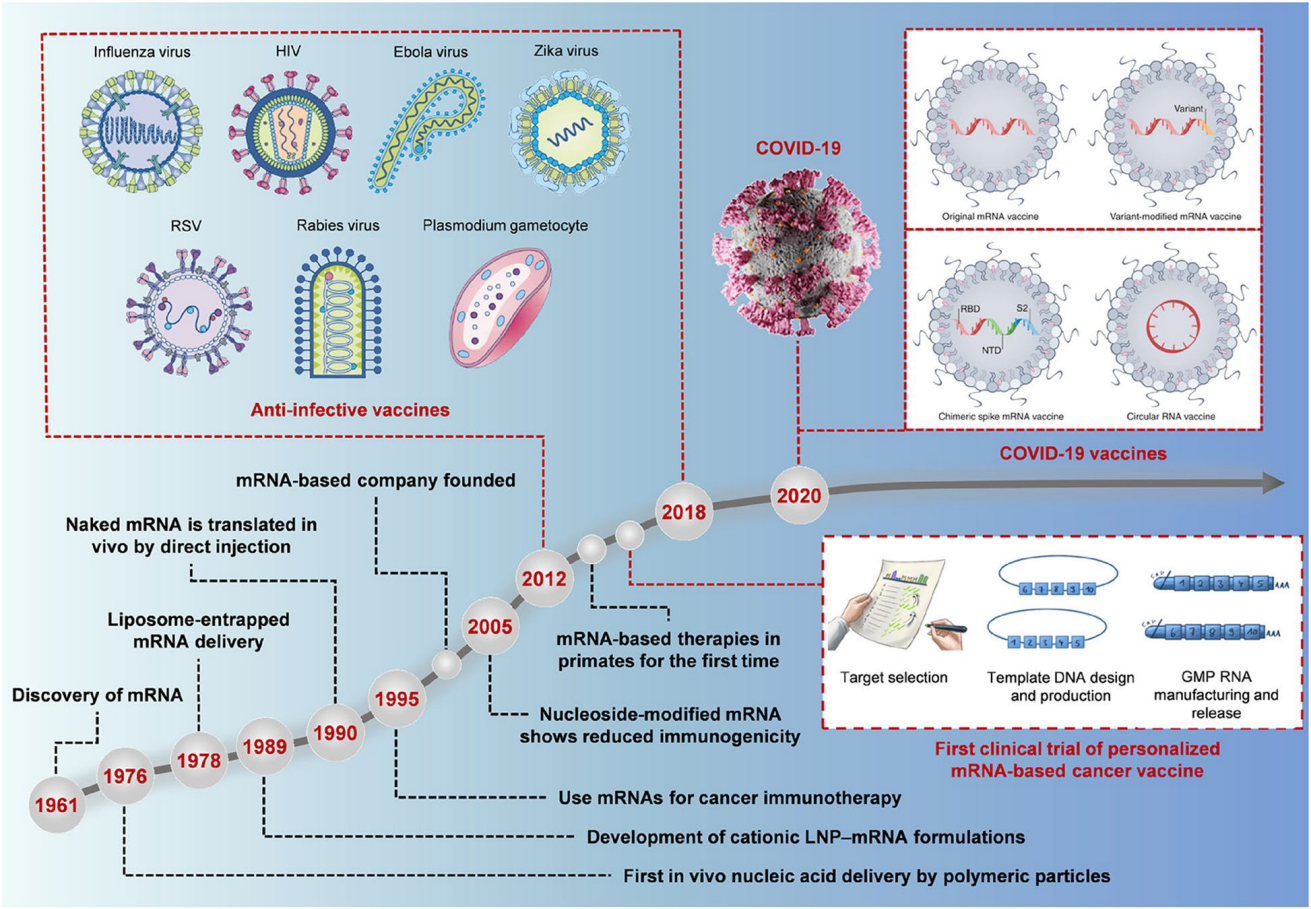
Timeline of some key discoveries for mRNA therapeutics development. Reproduced with permission [6]. Copyright 2017, Macmillan Publishers. Reproduced with permission [9]. Copyright 2023, American Chemical Society. Reproduced with permission [10]. Copyright 2022, Springer Nature. Reproduced with permission [5]. Copyright 2021, Springer Nature

 In the 1990s, Wolff et al. found for the first time that injecting in vitro transcription (IVT) messenger ribonucleic acid (mRNA) could produce proteins successfully in mice [1]. Since this discovery, the prospective of therapy based on mRNA has come into public view (Fig. 1). Benefited from the demand for coronavirus disease (COVID-19) vaccines, mRNA therapy is evolving rapidly [2, 3]. As a preventive or therapeutic drug, mRNA produces functional proteins with almost all known sequences, favorable safety, and effectiveness at any target, which has broad prospects in the fields of precision and personalized medicine [4–6]. However, due to its single-stranded structure, naked mRNA is destabilized in vivo and easily degraded by ribonuclease (RNase) [7]. Moreover, it is hard for a negatively charged mRNA macromolecule to cross the host cell membrane, which is also negatively charged, resulting in inefficient cell permeation [8]. To address these challenges, the rapid development of mRNA engineering technologies, including chemical modification, and the use of reasonable carriers to protect mRNA from RNase degradation and assist with intracellular mRNA delivery.

Nanoparticle (NP)-based platforms are widely considered the most promising potential mRNA drug delivery system (DDS) owing to its ability to alter the properties through controllable and simple chemical synthesis, resulting in enhanced mRNA-binding affinity and delivery potency [11]. The premise of mRNA therapy is to deliver mRNA to specific organs and cells accurately. However, the physiological structures and microenvironment vary considerably in different organs and cells, which challenges the precise delivery of mRNA NPs. Moreover, the in vivo biological barriers, the rapid clearance by mononuclear phagocytic system (MPS), and suboptimal biodistribution also influence the delivery of mRNA NPs [12].

To address these issues, researchers designed targetable mRNA NPs and successfully delivered mRNA to organs and cells through rational administration routes and specific design strategies. For instance, Lokugamage et al. [13] designed mRNA NPs targeting the lungs via intranasal administration for protection against influenza A virus. Due to the design of high molarity polyethylene glycol (PEG) and cationic helper lipids, mRNA NPs overcame the physiological barriers to reach the lung epithelial cells and released mRNA to prevent influenza A virus infection. Similarly, Yang et al. [14] designed hepatocyte nuclear factor 4 alpha (HNF4A)-mRNA NPs to target hepatocytes via intravenous administration based on the characteristics of abundant blood flow and endothelial fenestrations in liver. This brings new hope to the treatment of liver fibrosis.

Typically, vaccine administration routes, along with physiological characteristics of the target, affect the design of targeting strategies and mainly depend on the location and physiological characteristics of target organs/ cells. The different targeting strategies of mRNA NPs include passive, active, and endogenous targeting, which has shown variable influence on the distribution of mRNA NPs in vivo on systemic administration [15]. Passive targeting is usually affected by the physicochemical properties of NPs such as size, zeta potential, and pKa, while active targeting is mainly achieved by introducing target-specific ligands like antibodies and small molecules [16]. Notably, for endogenous targeting, the biomolecular corona (primarily protein corona) endows mRNA NPs with a new identity, which may mask the surface physicochemical properties of NPs and affect the targeted delivery of mRNA NPs in a way different from those of traditional passive and active targeting techniques [17]. Considering the complex microenvironment of different organs and difficult transfection characteristics of certain cells, the precise targeted delivery of mRNA NPs, especially extrahepatic targeting, requires careful and comprehensive design. However, few published reviews have focused on systems of mRNA targeted delivery to different organs and cells based on targeting strategies and administration routes.

In this review, we first introduce the chemical modifications of mRNA and the profiles of mRNA NPs, including lipid NPs (LNPs), polymers, and peptides. Second, we summarize the targeting mechanisms and cellular uptake processes that affect the delivery of mRNA NPs to different organs and cells. Third, we describe the structures of important organs and cells and the barriers to NP-based targeted delivery. These organs include the liver and other major extrahepatic organs such as the lung, spleen, brain, and fetus. Most importantly, we focus on the specific design of targeting strategies of mRNA NPs to overcome these barriers. Fourth, we detail the influence of administration routes on targeting design. In addition to the frequent routes of mRNA NPs, such as intravenous or intramuscular injection, we also discuss the intranasal administration and some new or unusual delivery routes, such as needle-free delivery, uterine injection, and vaginal atomization. Finally, we describe the challenges and opportunities for the development of mRNA NPs in the future.

## mRNA: chemical modifications

**Fig. 2:**
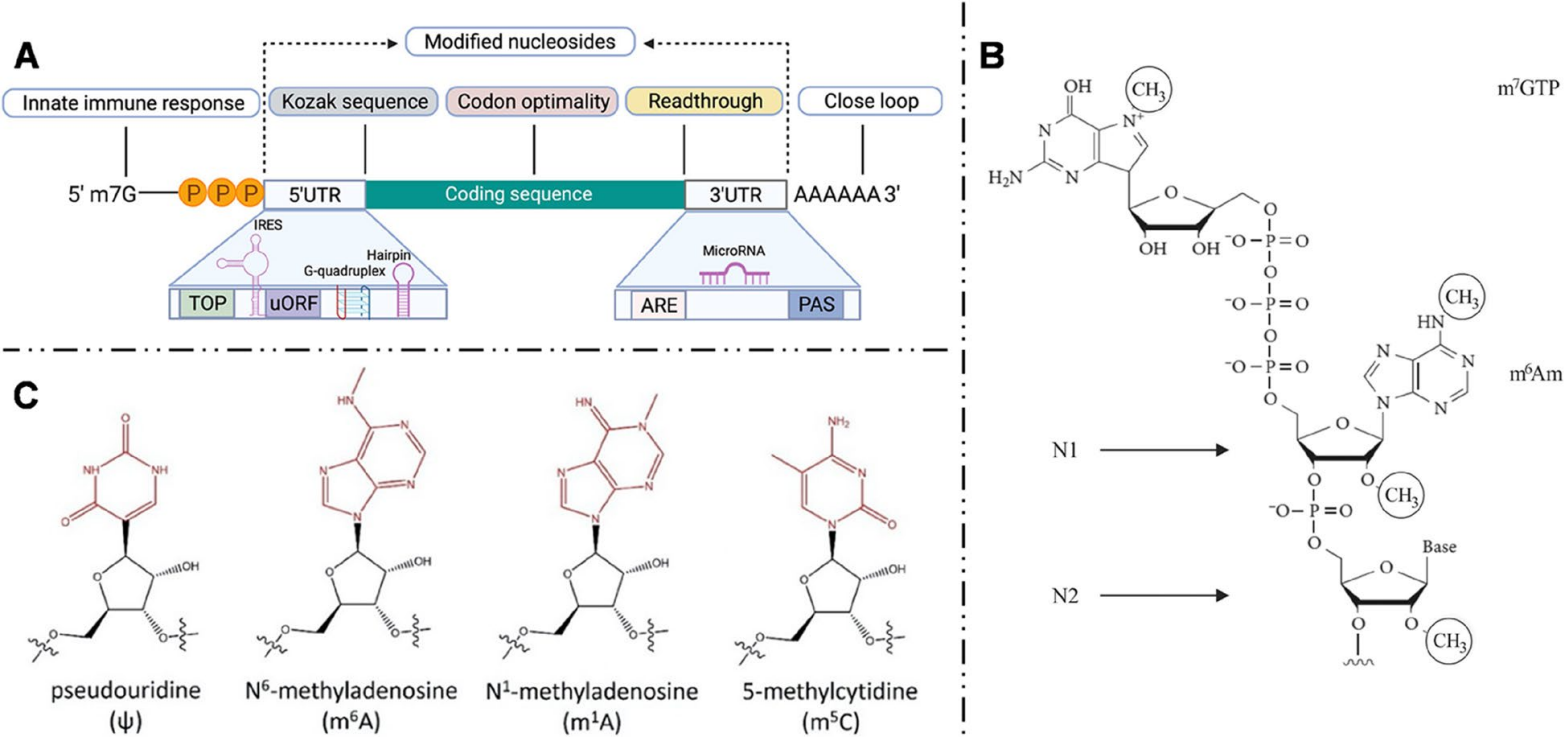
** A** Schematic representation of the IVT-mRNA and key structural elements. Reproduced with permission [26]. **B** 5′ cap structure of mRNA. Circles indicate the methyl group. Cap-1 and Cap-2 structures are expressed at N1 and N2 position, respectively. Reproduced with permission [26]. **C** Chemically modified nucleosides structures. Reproduced with permission [30]

The basic structure of IVT-mRNA closely resembles that of eukaryotic mRNA, encompassing five primary modifiable structural components: a 5′ cap, a 3′ poly(A) tail, 5′- and 3′-untranslated regions (UTRs), and an open reading frame (ORF) [5] (Fig. 2A). Structural modifications of mRNA are pivotal for enhancing its stability, attenuating immunogenicity, and bolstering translation efficiency. Researchers have elucidated numerous optimization strategies concerning mRNA structural elements.

### 5′ cap

The 5′ cap is a distinctive modification present at the 5′ end of most eukaryotic mRNA molecules [18]. Typically comprising of a 7-methylguanosine, it’s connected in reverse orientation to the first nucleotide via a 5’-5’ triphosphate linkage (Cap-0) [19]. The 5′ cap plays crucial roles in the translation, stability, reducing the immunogenicity and resistance to cellular exonucleases of mRNA [20, 21]. The Cap-0 structure provides steric hindrance that curtails mRNA degradation by nucleases and orchestrates translation initiation through its interaction with the eukaryotic initiation factor 4E (eIF4E) [22]. In addition to Cap-0, the methylation of 2’- hydroxyl groups of the inaugural nucleotide and the second nucleotide at the 5’ end of mRNA, namely Cap-1 and Cap-2 (Fig. 2B), has been proved instrumental in attenuating immunostimulatory responses and accentuating both the translation efficiency and stability of mRNA, often exhibiting superior translation efficacies relative to the conventional Cap-0 [5, 23].

### 3′ poly(A) tail

The 3′ poly(A) tail also plays a pivotal role in bolstering mRNA stability and facilitating protein translation [24]. This tail engages in interactions with a variety of proteins, notably poly(A) binding proteins (PABPs), which subsequently regulate mRNA translation and stability [20]. While the length of the 3′ poly(A) tail correlates positively with translation efficiency, an excessively long tail can compromise plasmid stability [25, 26]. Hence, a typical poly(A) tail is designed as 100–120 nucleotides long to harmonize translation efficiency with stability [27]. The precise length is often tailored based on target cells and the specific delivery environment.

### 5ʹ‑ and 3ʹ‑UTRs

UTRs are two non-coding segments of mRNA molecules located upstream (5’-UTR) and downstream (3’-UTR) of the ORF. The 5′- and 3′-UTRs play pivotal roles in regulating mRNA stability, translation efficiency, subcellular localization, and interactions between mRNA and its binding proteins [28, 29]. UTRs from highly expressed genes, such as the α- and β-globin genes, are the preferred choices for IVT-mRNA. Notably, the COVID-19 vaccines BNT162b and mRNA-1273 utilize the human α-globin gene for their UTRs [30]. However, the efficiency of UTRs varies depending on the target cell, necessitating specific optimization. It is imperative to note that an overly stable 5′-UTR secondary structure can hinder ribosomal binding to mRNA, while an excessively long 3′-UTR sequence can compromise translation efficiency [26].

### ORF

The ORF denotes the segment of mRNA responsible for protein coding. Comprised of continuous codons between the start and stop codons, the ORF dictates the amino acid sequence of the resultant protein, directly influencing mRNA stability and translation efficiency [31]. Given the differential codon preferences among species, codon optimization becomes essential when expressing heterologous proteins via IVT-mRNA. This can be achieved by substituting rare codons with common codons encoding the same amino acid residues, thereby enhancing translation without altering the protein sequence [32]. While optimizing the ORF with common codons is an attractive approach to augment protein translation efficiency, caution is warranted. Certain proteins necessitate conditions of reduced translation efficiency for correct folding. In such cases, the deliberate selection of rare codons ensures effective protein folding [33].

### Chemical modifications to mRNA backbone

The incorporation of chemically modified nucleosides represents a primary strategy to reduce the immunogenicity of IVT-mRNA. Commonly chemical modification methods include pseudouridine (Ψ), N^6^-methyladenosine (m^6^A), 5-methylcytidine (m^5^C), and N^1^-methylguanosine (m^1^A) [30, 34] (Fig. 2C). Ψ stands out as the most extensively employed modification, with both in vitro and in vivo experiments demonstrating its significant enhancement of mRNA translation efficiency, while concurrently attenuating its immunogenicity [35]. The COVID-19 vaccines, BNT162b2 and mRNA-1273, both incorporate Ψ modifications. Besides, Andries et al. [36] found that mRNA modified with N^1^-methylpseudouridine (m^1^Ψ) either alone or in combination with m^5^C outperformed the current mRNA platforms modified with Ψ. These indicate that chemically modified nucleosides could emerge as the mainstream approach for future treatments based on mRNA, producing satisfactory clinical outcomes. However, excessive modifications of nucleotides might interfere with the binding of other components to mRNA [37], potentially affecting mRNA translation. This aspect should be taken into consideration when modifying IVT-mRNA.

## mRNA NPs: advanced nanodelivery systems

**Fig. 3:**
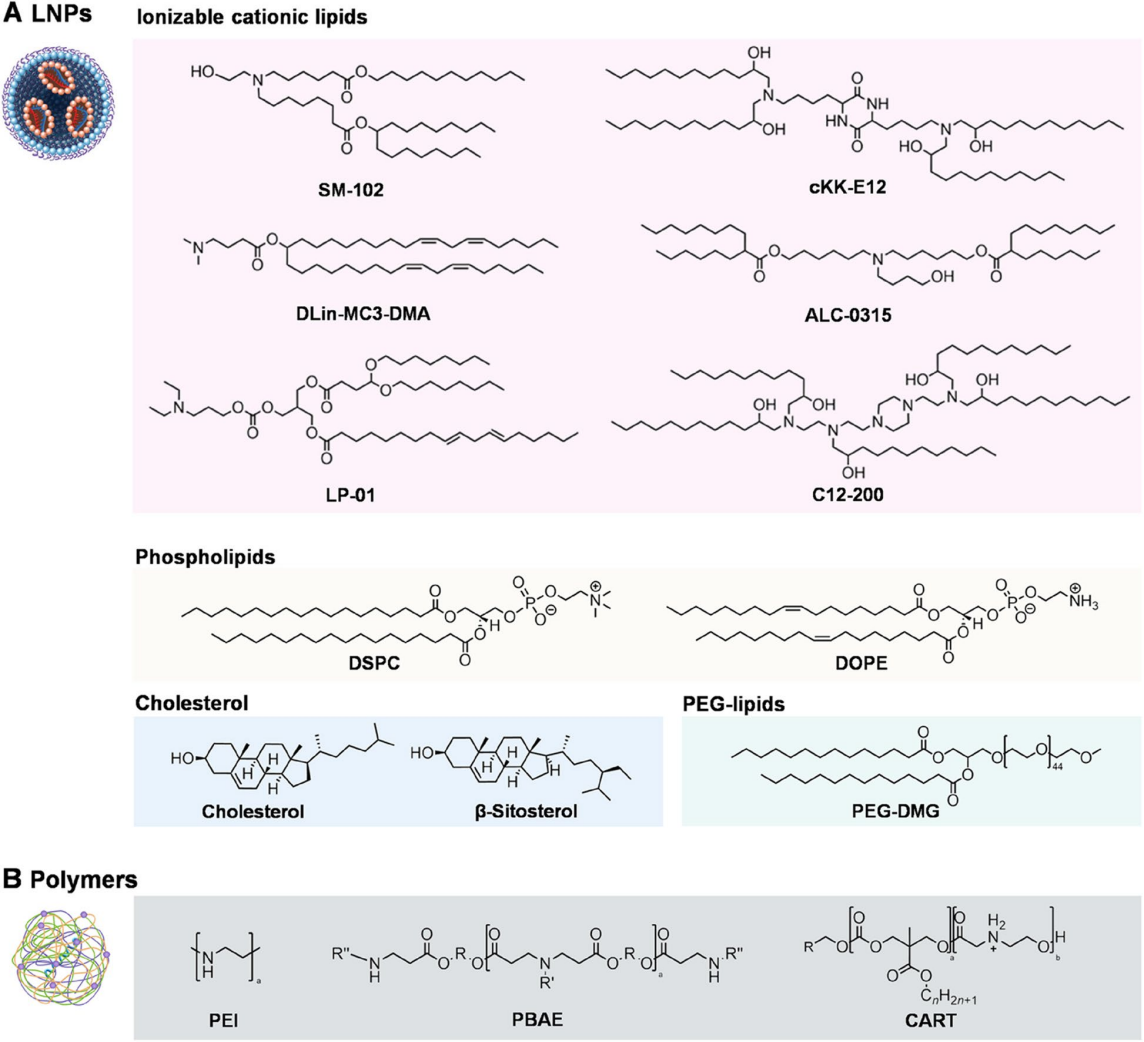
Self-assembled nanoparticles for the delivery of mRNA represented by lipid nanoparticles (LNPs) and polymers. **A** Most common chemical structures of LNPs formulation and **B** Polymers

Choosing suitable nanocarriers is a necessary step in the design of targeted delivery strategies. Generally, the optimal mRNA delivery nanocarriers must contain following characteristics. Primarily, the carriers should efficiently encapsulate mRNA and have stable circulation in vivo to protect mRNA against degradation by nucleases. Secondly, the mRNA carriers need target the specific organ or cell, to facilitate cellular uptake and intracellular release. Furthermore, the carriers must be biocompatible, low-priced, and easily production. This section focuses on the non-viral NP delivery systems packaging mRNA synthesized in vitro, including lipid nanoparticles, polymers, and peptide/protein NPs (Fig. 3). Understanding NP characteristics is essential to optimize their delivery strategy to targeted organs and cells.

### LNPs

LNPs are the most researched and widely used mRNA DDS [38, 39]. Cationic lipids, such as 1,2-di-O-octadecenyl-3-trimethylammonium-propane (DOTMA) and 1,2-dioleoyl-3-trimethylammonium-propane (DOTAP) that contain positively charged quaternary ammonium groups and can effectively encapsulate negatively charged mRNA, were widely used in early research [40]. Despite exhibiting a promising effect in the development of vaccines for cancer [41] and experimental autoimmune encephalomyelitis [42], cationic lipids exhibit potential cytotoxicity and instability in systemic circulation, limiting their development and clinical transformation [43].

Through in-depth studies, researchers presented ionizable LNPs, consisting of ionizable cationic lipids, neutral helper lipids (phospholipids), sterol lipids (cholesterol), and PEG polymer–conjugated lipids (PEG-lipids) [44]. The use of this kind of LNPs has gradually increased and are being developed as advanced nanocarriers in mRNA DDS, including in COVID-19 mRNA vaccines [45].

#### Ionizable cationic lipids

Unlike traditional cationic lipids, ionizable cationic lipids remain neutral in blood circulation (physiological pH) and become positively charged by protonation in acidic pH. This pH-sensitive property confers multiple advantages to ionizable cationic lipids in terms of mRNA delivery. Firstly, lipids with positive charge interact with mRNAs in acidic conditions, thus increasing encapsulation efficiency. Secondly, ionizable cationic lipids remain stable in blood circulation and reduce toxicity. Additionally, reduced intracellular pH after cellular uptake causes protonation of ionizable cationic lipids, which aids in mRNA release. The common ionizable cationic lipids used in LNPs that entered the clinical trials are DLin-MC3-DMA (MC3) [46], cKK-E12 [47], C12-200 [47], SM-102 [48], ALC-0315 [49], and LP01 [50].

#### Phospholipids and cholesterol

Phospholipids have strong bilayer formation characteristics and high phase transition temperature, while cholesterol [51] has excellent membrane fusion ability. Both ensure structural stability of LNPs, regulate transfection efficiency, promote endosomal escape, and intracellular uptake of mRNA. Common phospholipids include 1,2-distearoyl-sn-glycero-3-phosphocholine (DSPC) [52] and 1,2-dioleoyl-sn-glycero-3-phosphoethanolamine (DOPE) [53].

#### PEG-Lipids

PEG-lipids are the least content components in LNPs, located on LNP surface. PEG-lipids play important roles in improving hydrophilicity, determining LNP size, preventing LNP aggregation to maintain stability, preventing LNPs from being quickly removed, and improving the circulating half-life of LNPs in blood [54]; hence, they are also called “stealth” lipids. However, the structure and quantity of PEG must be carefully adjusted to prevent inducing potential anti-PEG antibodies and decreased transfection efficiency [55].

### Polymers

Polymers are also widely used DDSs [56]. Polymers are generated by the self-assemblage of biodegradable amphiphilic block-copolymers [57]. Because the physicochemical properties of polymers, such as their size, structure, and function, are easily adjustable, and the efficacy, stability, and organ targeting capability of polymers are affected by the formula components, polymers have great potential in mRNA delivery [58]. As positively charged polymers are highly probable to combine with negatively charged mRNA by simple electrostatic interaction and transport them into the cell, the earliest mRNA-carrying polymer NPs were composed of cationic polymers containing amino groups such as polyethyleneimine (PEI) [59].

However, owing to the positive charge, like cationic lipids, cationic polymers have non-negligible cytotoxicity, limiting clinical development. Development of functional and biodegradable polymers is presently a hot spot to address toxicity issues. Examples of such polymers include polyester, poly(β-amino ester) (PBAE), poly(amine-co-ester) (PACE), poly(2-(dimethylamino)ethyl methacrylate) (PDMAEMA), poly(lactic-co-glycolic acid) (PLGA), and poly(lactic acid) (PLA) [60]. Polymers are used less frequently than LNPs in clinical practice for mRNA delivery, but their active chemical properties provide additional material characteristics, such as cell and tissue tropism (e.g., for lungs) and endosome escape ability [61], which are beneficial for organ-specific delivery and increases mRNA translation efficiency.

### Peptides

Peptides are biopolymers composed of amino acids. Cell-penetrating peptides (CPPs) are small molecular polypeptides with cell membrane permeability derived from various proteins [62]. CPPs with different functions can be constructed by optimizing the design of amino acids. CPPs can directly penetrate the cell membrane and act independent of the receptor, which is suitable for most cell types [63]. Further, CPPs can electrostatically bind mRNA to form nanocomposites. Thanks to the CPP membrane-penetrating properties, mRNAs are efficiently delivered, especially into antigen-presenting cells (APCs) to induce an immune response [64]. CPPs commonly used for mRNA delivery include RALA peptide and PepFect14 [65, 66]. Using CPPs for mRNA delivery is still in its infancy, but the prospect is broad.

### Hybrid NPs

Hybrid NPs composed of two or more materials, with excellent physicochemical characteristics, have been the subject of research lately. Several combinations have been applied for mRNA delivery, including the following:

#### Polymeric lipid hybrid NPs (PLNs)

PLNs usually consist of a polymer-mRNA core and a lipid-PEG shell, which are promising delivery vehicles. PLNs have the physical stability and biocompatibility of both polymer and lipid NPs [67]. Among them, polymers can control drug release, while lipids can improve the stability of NPs in serum circulation [68]. For example, combining hydrophobic polymers, ionizable lipids, and PEG-lipids improves serum stability, showing potential for mRNA therapy of tumors [69]. Moreover, simple optimization or functionalization of PEG-lipid shell terminals can help achieve local- or organ-targeted delivery, making PLNs an ideal mRNA delivery platform.

#### Organic/inorganic hybrid NPs

Inorganic materials present a promising gene delivery vector due to their commendable biocompatibility, tunable physicochemical properties, satisfactory stability, and cost-effectiveness [70]. Common inorganic nanomaterial platforms encompass gold-based NPs, silica-based NPs, calcium phosphate-based NPs, graphene-based NPs and metal-organic framework NPs. However, within the realm of mRNA delivery, inorganic nano-platforms remain in the early stages of development. Inorganic materials are often combined with cationic polymers for mRNA delivery into host cells. Cationic polymers such as PEI improve the binding between NPs and mRNA, thus enhancing mRNA transfection and proton sponge effects [71]. In turn, inorganic materials reduce the cytotoxicity of cationic polymers and favor the biocompatibility of NPs. Organic/inorganic hybrid NPs under study include graphene oxide-PEI (GO-PEI) [72], mesoporous silica-PEI [71], and zirconium-based metal-organic framework (MOF) functionalized with polycationic ethanolamine (EA) conjugated poly (glycidyl methacrylate) [MOF-PGMA(EA)] [73]. Additionally, researchers have proposed a lipid/calcium/phosphate (LCP) system [74]. Calcium phosphate was used as the core to promote intracellular mRNA release, with PEGylated DOTAP/DOPE liposome as the shell to maintain NP stability.

### Other NPs

#### Stimuli-responsive NPs

Stimuli-responsive NPs refer to the drug release under certain stimulus conditions, such as pH, reductant, and other special biomolecules [75]. The versatility of both the chemical compositions and surface functional groups of NPs enables controlled mRNA release through suitable biodegradation or stimulus activation mechanisms [76]. Currently, majority research tends to use the low pH characteristics of lysosomes to open the nano-shell and release mRNA [77, 78]. Other characteristics of target cells, such as intra/extracellular adenosine triphosphate (ATP) concentration gradient and glutathione content, have been proposed to be used to trigger the cracking of smart nano-shells. Yoshinaga et al. synthesized an ATP-responsive polyplex micelle type nanocarrier loaded with mRNA [79]. It can release mRNA in high ATP concentrations due to its special phenylboronate ester crosslinking structure. Moreover, a glutathione-responsive nanoplatform in the form of cationic block-copolymer with imidazole residues and disulfide bonds showed efficient transfection and good biocompatibility in multiple cell types [80]. Given the vastly different organ and cellular states under disease conditions, comprehensive in vivo investigations are required to evaluate the therapeutic potential of stimuli-responsive mRNA NPs for various disease models.

#### Biomimetic synthetic NPs

Besides the previously mentioned vectors, a novel aspect of mRNA targeted delivery is the use of biomimetic synthetic NPs [81]. Materials such as LNPs, polymers, or inorganic materials with specific bonding characteristics are used to package the core, whereas the surface is coated with a cellular membrane [82]. Different tissues can be targeted by changing the type of cellular membrane. Red blood cells (RBCs) were the first to be included in the study of biomimetic coatings [83]. Since then, various types of cell membranes have been studied to wrap NPs, including stem cells [84], macrophages [85], and cancer cells [86]. Besides, a variety of cell membrane hybrid coatings have been designed to develop NPs with multiple biological functions. Cell membrane coatings, whether single or hybrid, protect NPs better than the traditional ligand-receptor binding strategies during systemic circulation or drug-accumulated lesion. Therefore, these materials may define the future direction of mRNA vaccine development.

## mRNA NPs: targeting and endocytic mechanisms

Successfully delivering mRNA NPs to their intended target sites is crucial for effective mRNA therapy. Targeting mechanisms that determine how NPs are recognized and absorbed by specific organs or cells include passive, endogenous, and active targeting. Synergistic interactions may occur among these targeting mechanisms to enhance the accumulation and bioavailability of NPs at target sites. Endocytosis is the key pathway for NPs to enter cells, and understanding these endocytic mechanisms can help optimize NP size, shape, and surface properties for efficient cellular uptake and intracellular release [87]. This section will provide a detailed overview of the targeting and endocytic mechanisms, offering a theoretical basis for the design of mRNA NP targeting strategies.

### Targeting mechanisms

**Fig. 4:**
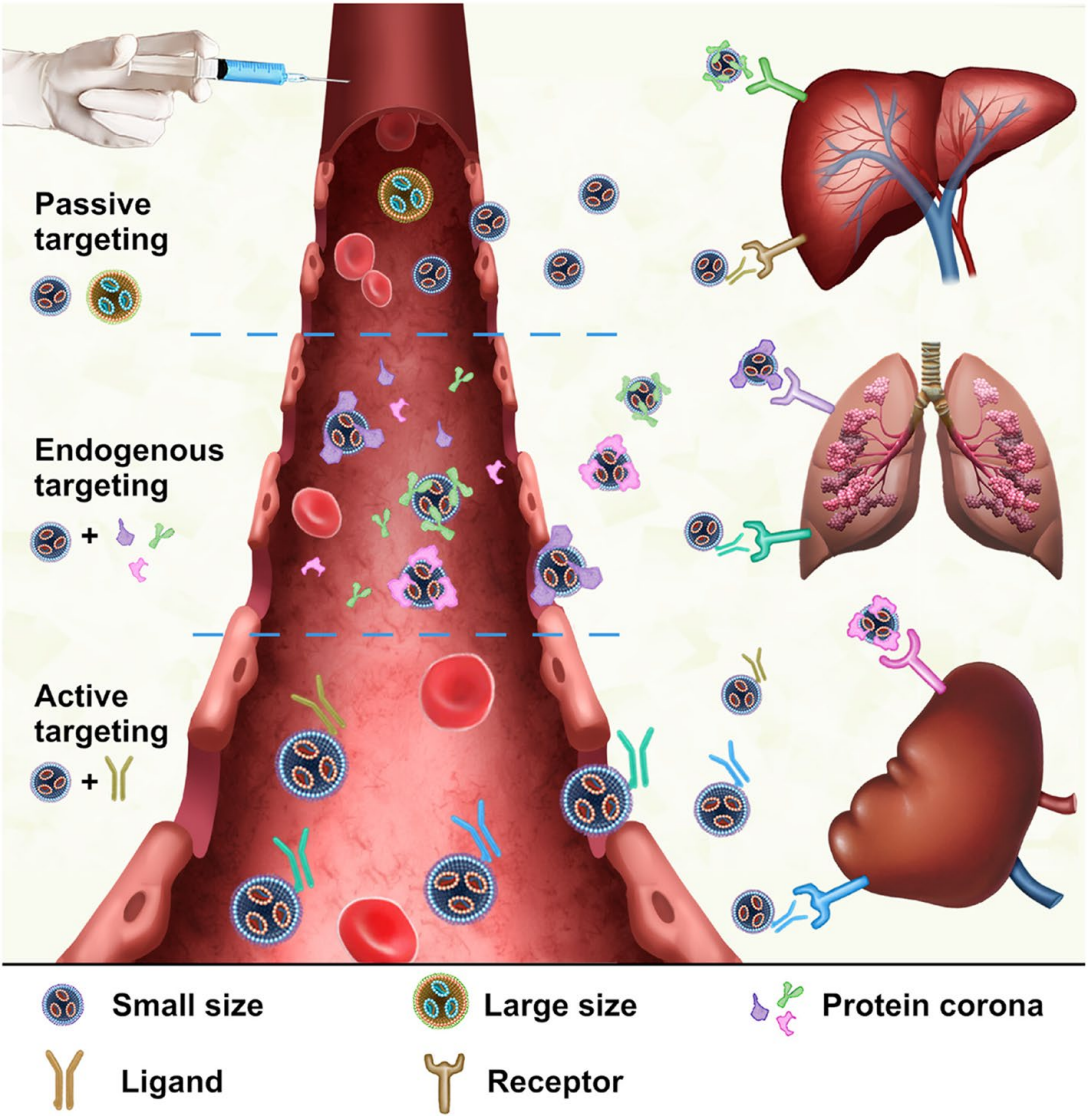
Schematic illustration of targeting mechanisms of mRNA NPs. After intravenous injection, NPs achieve specific delivery to organs through passive, endogenous and active targeting. Passive targeting involves adjusting the size, structure and other physicochemical properties of NPs to achieve targeted delivery of mRNA. Endogenous targeting involves binding to different subsets of plasma proteins, guiding NPs to specific organs and being absorbed by target cells. Active targeting relies on modifying the NP surface with specific ligands that can specifically bind to the receptors highly expressed by target cells

After mRNA NPs enter the systemic circulation, different biological processes affect the final biological distribution of mRNA in vivo. Precision delivery of mRNA NPs to target organs and cells is a prerequisite to maximize pharmacodynamic effect. Targeted therapy can significantly increase NP concentration in the treatment site and reduce the dosage and systemic toxic side effects [88]. The targeting strategies of mRNA NPs include passive targeting, endogenous targeting, and active targeting (Fig. 4). All of them should be considered in the targeting design of mRNA NPs.

#### Passive targeting

Passive targeting is an involuntary behavior to enter specific organs, tissues, and cells without relying on the recognition ability of target molecules by adjusting the size, morphology, structure, surface, and other physicochemical properties of mRNA NPs. Passive targeting is closely related to the anatomical structure and physiological characteristics of the target organ, which is usually achieved through the “permission” of biological structure or cell uptake and some external conditions, such as magnetic field, electric field, or special drug delivery route. For example, discontinuous blood vessels in the liver allow mRNA NPs of a certain size to penetrate [89], or the drugs can target the lungs by inhalation. The structure and characteristics of various organs and cells will be introduced in detail in the fourth section.

#### Endogenous targeting

Endogenous targeting is a special passive targeting, which mainly means that NPs will combine with different biomolecules in systemic circulation, to form a specific “biomolecular corona”, especially with protein in blood to form a “protein corona,“ which can be transported to target organs or cells without targeting ligands [90]. During in-depth exploration of the relationship fate between protein corona and NPs in vivo, the influence of protein corona on mRNA NP targeting was found to have two sides. The adsorption process of protein coronas is known to be difficult to control, and protein coronas can even mask the targeting ligands, leading to loss of targeting efficacy. Based on these results, the protein corona is considered an “enemy” in NPs targeting strategies, necessitating resistance to protein corona adsorption in vivo. However, emerging evidence shows that some specific protein coronas in the blood can improve mRNA NP delivery to specific tissues [91]. For example, apolipoprotein E (ApoE) is associated with liver-specific delivery of NPs. If these protein coronas with specific targeting properties can be reasonably utilized, the targeting strategies of mRNA NPs can be greatly optimized.

#### Active targeting

The off-target effects of passive targeting are inevitable. To ensure selective delivery to organs or cells, various active targeting strategies are often employed in conjunction. mRNA NPs rely on the recognition capabilities of targeting molecules to access specific organs and cells. By modifying the NP surface with chemical or biological components, the NPs specifically bind to host cell receptors or biomarkers, thereby enabling precise identification and delivery to target cells or organs. For example, NPs modified with N-acetylgalactosamine (GalNAc) actively target the asialoglycoprotein (ASGPR) receptors on hepatocytes, which is the best elucidated clinical example till date [92]. Common active targeting ligands that modify delivery systems include antibodies, aptamers, peptides, protein and lipid molecules, polysaccharides, and vitamins. mRNA NPs functionalized with various ligands can undergo receptor-mediated endocytosis in specific cells. To enhance targeting accuracy, mRNA NP surfaces can be designed with multiple ligands to optimize endocytosis and binding to target cells.

### Endocytic mechanisms

**Fig. 5:**
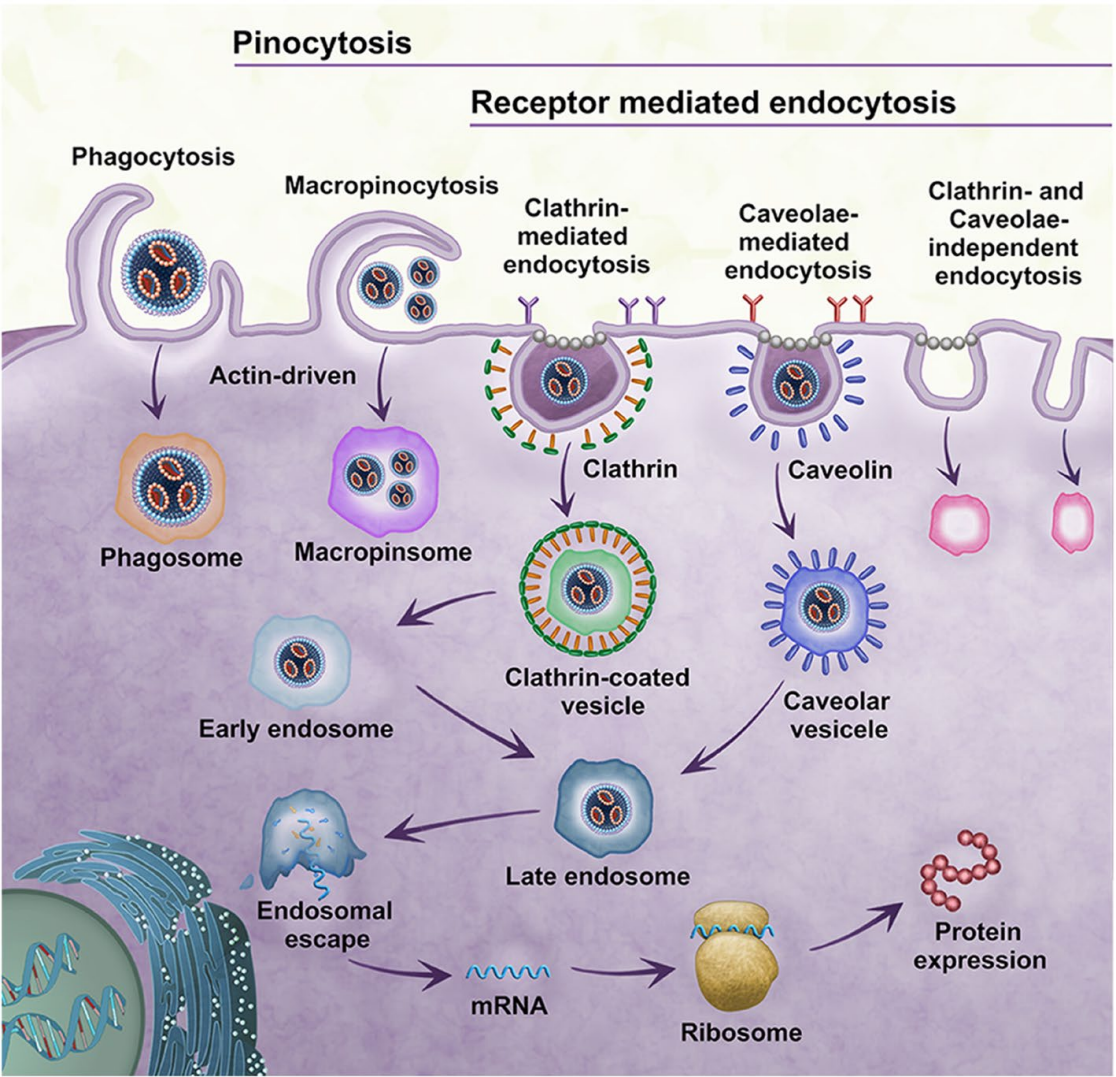
Schematic illustration of endocytosis patterns of mRNA NPs. mRNA NPs enter cells via five pathways, including phagocytosis, macropinocytosis, clathrin-mediated endocytosis, caveolar-mediated endocytosis and clathrin-/caveolar-independent endocytosis and undergo different intracellular fates

mRNA translation occurs in the cytoplasm, so the mRNA vectors must be able to cross the cell membrane and internalize to the cytoplasm. Cell membrane is a powerful obstacle to intracellular transport, which affects the uptake of NPs by host cells [93]. Generally, the mRNA-carried NPs pass through the cell membrane via the endocytosis, and release mRNA from endosomes and lysosomes to start translation and produce protein. Endocytosis mainly includes phagocytosis and pinocytosis (Fig. 5).

#### Phagocytosis

Phagocytosis is the prominent function of some immune cells, which can also transpire to a lesser degree in non-professional phagocytes such as endothelial cells or liver cells [94, 95]. This process, driven by actin, engulfs mRNA NPs. Phagocytosis mainly absorbs NPs larger than 250 nm, however with the in-depth research, evidence demonstrated that phagocytosis could also act on NPs with a diameter of 100 nm or smaller [96].

#### Pinocytosis

Pinocytosis is suitable for all types of cells, taking up small particles that phagocytosis cannot. There are four mechanisms of pinocytosis: macropinocytosis, clathrin-mediated endocytosis (CME), caveolae-mediated endocytosis (CvME), and clathrin-/caveolae-independent endocytosis [96].

Macropinocytosis is an endocytosis process driven by actin. Unlike in the phagocytosis, macropinocytosis seems to be non-selective, thus supplementing other endocytosis routes of drug carrier uptake, especially disregarding the invisible coating on the surfaces of NPs (such as PEG) [97].

CME is a receptor-dependent endocytosis, which widely exists in cells and is also the major pathway to internalize mRNA NPs, leading endogenous and active targeting. Related receptors include low-density lipoprotein receptors (LDLRs) and transferrin receptors. CME is a key approach in the targeting design of mRNA nano-drugs, especially of surface ligand modification [98].

CvME is receptor-dependent pinocytosis as well, which exists in lots of specific cells, such as fibroblasts, smooth muscle cells, and endothelial cells [99]. CvME is related to caveolin (a dimer protein that binds cholesterol) and involved in some critical biological processes in cells [100].

There are other small structures on the plasma membrane named “lipid rafts,” which enable clathrin- and caveolae-independent endocytosis, yet its mechanism is not clear yet. It is worth noting that for mRNA NPs, CME, CvME, or multiple endocytosis may contribute to their intracellular internalization [101].

## mRNA NPs: targeting design strategies for different tissues

mRNA drugs must generate enough encoded proteins within the target cells to achieve preventive or therapeutic effects. Consequently, the efficacy of mRNA therapy is closely related to specific administration routes and target organs/cells. In this section, we will elaborate on the distribution characteristics of mRNA NPs in organs and the potential barriers they may face before reaching the target cells, discussing the impact of targeting mechanisms and cellular endocytosis pathways on targeting strategies of mRNA NPs. Particular emphasis is placed on targeting liver, extrahepatic organs (such as lung, spleen, brain, bone, eye, heart, and fetus) and the cellular-level design strategies for mRNA NPs to identify their key parameters with high selectivity and specificity towards target organs and cells and implement precise mRNA NP targeting in vivo.

### Liver

**Fig. 6:**
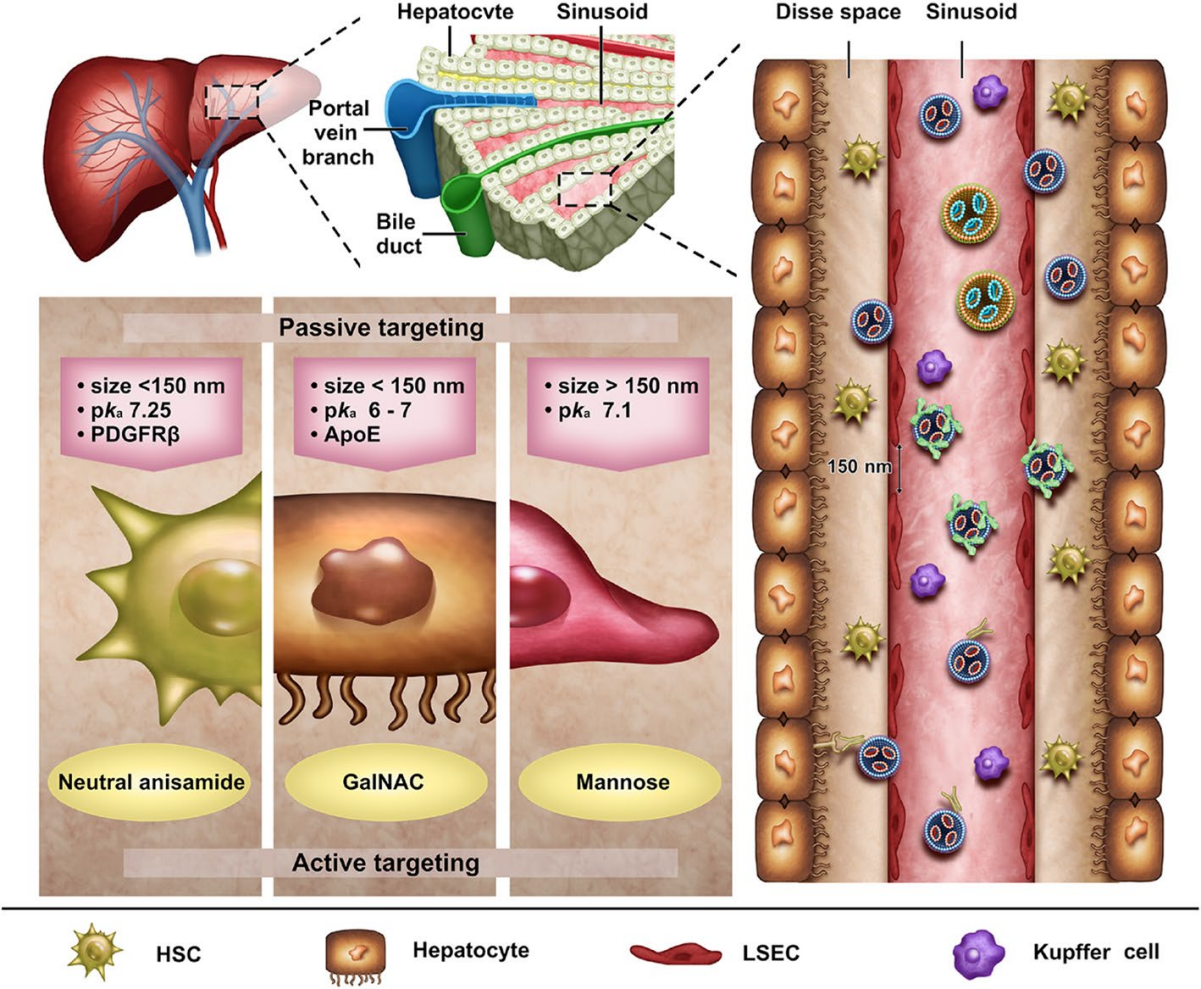
Schematic illustration of the structure of the liver and the journey of mRNA NPs to all kinds of cells in the liver via intravenous injection. The passive and active targeting characteristics of hepatocytes, hepatic stellate cells and hepatic endothelial cells are also presented

The liver is the major organ for protein synthesis and immune defense. Following intravenous injection, mRNA NPs tend to accumulate in liver, mainly due to its abundant blood supply, low flow velocities, and unique fenestrated type of hepatic sinusoidal endothelium (Fig. 6) [102]. Hepatic sinusoidal endothelium differs from the endothelia in other organs, with a fenestrae range of approximately 150 nm but lacking a basement membrane [89]. Consequently, hepatic sinus endothelium can act as an effective “ultrafiltration system” or “sieve,“ allowing small-sized NPs to enter the Disse space where hepatocytes and hepatic stellate cells (HSCs) are located without restriction, and thereafter deliver mRNA into hepatocytes and HSCs. Conversely, large NPs will be absorbed by Kupffer cells (hepatic macrophages) around the hepatic sinus. This suggests that the size of mRNA NPs is an important factor that affects cellular targeted delivery. Moreover, the adhesion of protein coronas in blood is also a critical factor in the preferential accumulation of mRNA NPs in liver. Despite some progress made in hepatic targeted mRNA delivery through nanocarriers, challenges still exist in successfully delivering mRNA to specific liver cells. Accordingly, this section focuses on the specific cell types in the liver and, based on the LNP and polymer category, provides a comprehensive discussion of the targeting strategies for delivering mRNA to different cell types (Table 1).

**Table 1:** Summary of liver-targeted LNP-based mRNA delivery strategies

Delivery systems	Formulation (mol%)	Administration route	Targeting features	Properties of mRNA NPs			Target cells	Ref.
				Size (nm)	Zeta (mV)	pKa		
***Passive targeting***								
LNP	MC3: DSPC: Chol: DMPE-PEG_2000_ 50: 10: 38.5: 1.5	i.v.	Size Surface composition of LNP	65	/	6.44	Hepatocytes	[46]
LNP	5A2-SC8: DOPE: Chol: DMG-PEG 23.81: 23.81: 47.62: 4.76	i.v.	Adjust the proportion of ionizable cation and zwitterionic phospholipid	95-101	-3.58	6.67	Hepatocytes	[103]
LNP	3060_i10_: DOPE: Chol: C_14_-PEG_2000_ 35: 16: 46.5: 2.5	i.v.	pKa	124.6	0.43	6.40	Hepatocytes Kupffer cells Endothelial cells	[104]
LNP	CLA: DOPE: Chol: DMG-PEG 31.25: 23.44: 39.06: 6.25	i.v.	CLA	100-150	Neutral charge	/	/	[105]
LNP	5A2-SC8: DOPE: Chol: DMG-PEG: DODAP 19: 19: 38:4: 20	i.v.	20% ionizable cationic lipid DODAP ApoE	122.4	**/**	6.46	Hepatocytes (93%)	[106]
LNP	cKK-E12: DOPE: 20α-OH: C_14_-PEG 35: 16: 46.5: 2.5	i.v.	ApoE LDLR	70.1	**/**	/	Hepatocytes Kupffer cells DCs	[91]
LNP	cKK-E12/A6: DOPE: Chol: C_14_-PEG_2000_ 35: 16.0: 46.5: 2.5 (A molar ratio of cKK-E12:A6 at 7:3)	i.v.	Albumin-associated macropinocytosis and endocytosis Introducing alkyne and ester groups into the lipid tails of MC3	Fluc mRNA: 99.5 ± 0.3 hEPO mRNA: 91.2 ± 0.5	-2.1 ± 1.3	6.78 ± 0.14	Hepatocytes	[107]
LNP	MC3: DSPC: Chol: PEG-lipid 50: 10: 38.5: 1.5	i.v.	ApoE	/	/	/	Hepatocytes in liver fibrosis	[14]
LNP	CL15A6: DOPE: cholesterol: DMG-PEG_2000_ 50: 20: 30: 3	i.v.	pKa Clathrin-mediated endocytosis through PDGFRβ receptors	179.2 ± 9.04	-9.6 ± 6.3	7.3 ± 0.11	HSCs(80%)	[108]
LNP	MC3: DSPG: Chol: PEG-DMG 50: 10: 38.5: 1.5	i.v.	Anionic LNP Stabilin receptors	66.6 ± 22.0	-20	/	LSECs	[109]
LNP	cKK-E12: DOPE: 20α-OH: C_18_PEG_2000_ 50: 10: 38.5: 1.5	i.v.	Modified cholesterols	80.1	/	5.6	LSECs Kupffer cells	[110]
***Active targeting***								
PLN	DOTAP: CHEMS: Chol: PEG_2000_ + GalNAPc-targeted polymer	i.v.	Active targeting (GalNAc ligand)	OTC mRNA/LNP: 65.4 ± 5.8 Polymer micelle: 15.5 ± 0.3	LNP: 0.84 ± 0.71 Polymer: 0.78 ± 0.91	/	Hepatocytes	[111]
LNP	AA-T3A-C12: DSPC: Chol: C_14_-PEG_2000_ 50: 10: 38.5: 1.5	i.v.	Active targeting (Anisamide-tethered lipidoids)	<100	neutral charge	5.72	HSC Hepatocytes	[112]
LNP	246C10: DOPE: Chol: C_16_-PEG_2000_ 26.5: 20: 50.5: 3.0 (2.5% mannose-PEG lipid)	i.v.	Active targeting (Mannose ligand)	117±11	-0.997 ± 0.21	6.75	LSECs	[16]
LNP	MC3: DSPC: Chol: DSPE-PEG_2000_-mannose 50: 10: 38.5: 1.5	i.v.	Active targeting (Mannose ligand)	153.2±1.1	-5.37 ± 2.40	/	LSECs	[113]

#### LNPs

LNPs are the most researched liver-specific targeting nanosystems loaded with mRNA. The main liver-specific targeting mechanisms are passive and endogenous targeting, owing to the structure and function of the liver. Research indicates that LNPs with a pKa value of six -seven are best-suited for mRNA delivery to the liver [114]. Among them, ionizable cationic lipids containing amino groups, such as DLin-MC3-DMA (MC3) [46], 5A2-SC8 [103], 306O_i10_ [104], and cationic lipid-modified aminoglycosides (CLAs) [105] exhibit high liver-targeting efficacy. The distribution of the LNPs with amino groups in liver can be as high as 81%, with the mRNA expression over 90% [104]. Nevertheless, the targeted precision delivery of mRNA should be carefully evaluated at the cellular level, as the degree of mRNA expression varies among different liver cell types in the above-mentioned research.

**Fig. 7:**
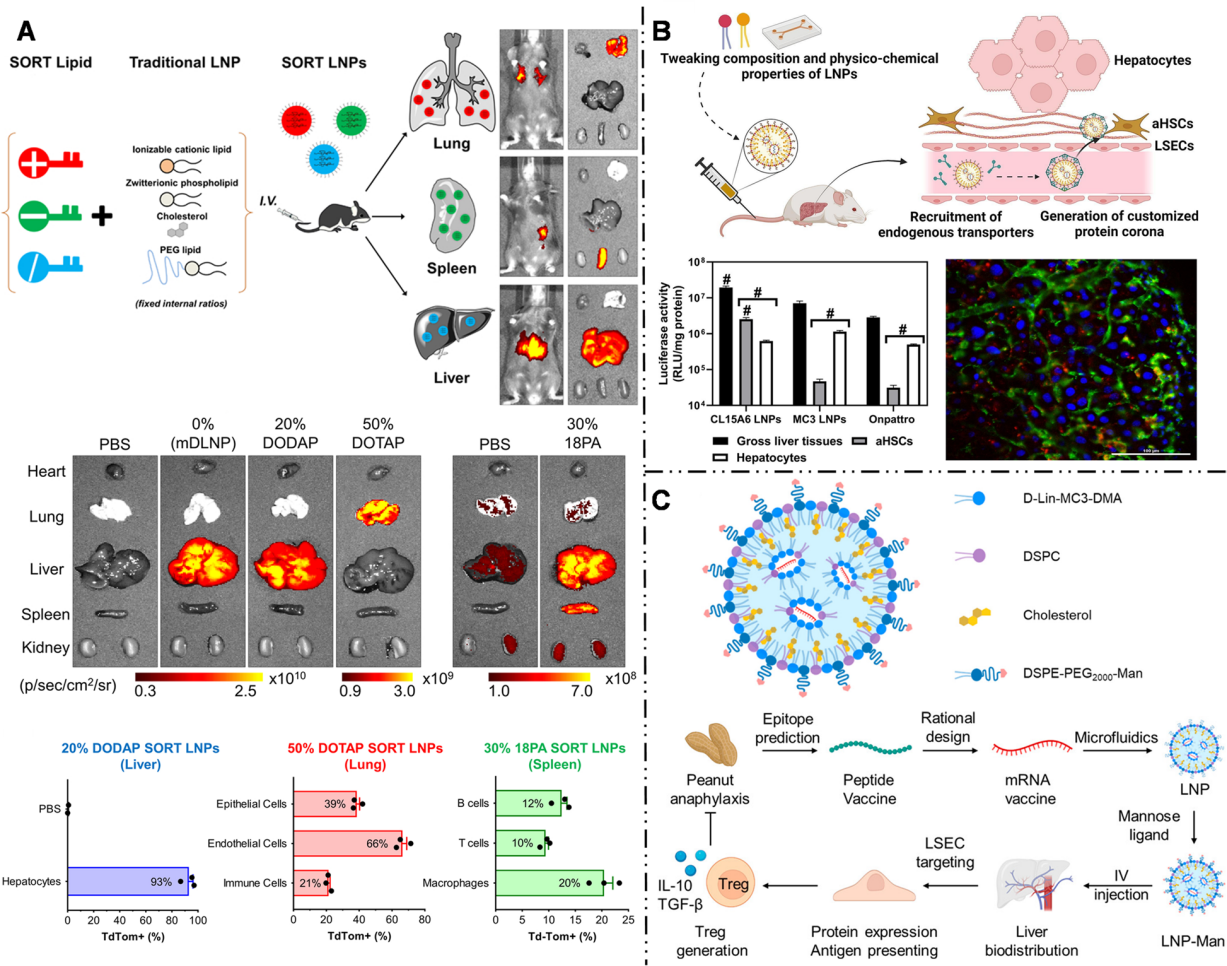
** A** Selective ORgan Targeting (SORT) strategy allowed mRNA-LNPs to precisely deliver mRNA into specific organs. Adding additional lipid ingredients (permanent cationic lipid, permanent anionic lipid or ionizable cationic lipid) to the traditional LNPs systematically changed the targeted delivery of mRNA-LNPs. Adding 20% ionizable cationic lipid (such as DODAP) enhanced the delivery of mRNA-LNPs to the liver, while adding permanent cationic lipid (50% DOTAP) and permanent anionic lipid (30% 18PA), the expression of luciferase was transferred to lung and spleen respectively. Reproduced with permission [106]. Copyright 2020, Springer Nature. **B** Optimized CL15A6 LNPs achieved successful transfection of over 80% of HSCs in vivo in liver fibrosis mice. The luciferase activity results revealed a 4-fold higher in the aHSCs compared to hepatocytes, and the CLSM showed mCherry fluorescence was scattered in the perisinusoidal area of liver microenvironment rather than being evenly distributed in the hepatocytes. Reproduced with permission [108]. Copyright 2023, Elsevier. **C** Mannose-modified mRNA-LNPs was designed for targeted delivery of mRNA-encoded peanut allergen epitopes to LSECs and modulating the immune response in mice. Reproduced with permission [113]. Copyright 2023, American Chemical Society

Hepatocytes account for about 80% of liver tissue and are implicated in many hereditary diseases, making them the primary targets for mRNA delivery. To enter the Disse space and directly contact hepatocytes, mRNA-LNPs for hepatocyte-targeted delivery are generally smaller than the fenestration of hepatic vascular endothelium, which measures approximately 150 nm. Hashiba et al. [115] synthesized a series of mRNA-LNPs with varying particle sizes and demonstrated that LNPs with sizes ranging from 60 to 100 nm can pass through the sinusoidal endothelial fenestrae of the liver, enhancing the hepatocyte targeting efficiency of mRNAs. Siegwart et al. [106, 116, 117] proposed a selector organ targeting (SORT) strategy (Fig. 7A). They synthesized 5A2-SC8-mRNA-LNPs with a particle size of approximately 122 nm. Incorporation of 20% ionizable cationic lipid 1,2-dioleoyl-3-dimethylammonium-propane (DODAP) into LNPs improved the uptake efficiency of hepatocytes to as high as 93% [106]. In subsequent studies, Siegwart et al. [116] elucidated the hepatocyte-targeting mechanism of 5A2-SC8-mRNA-LNPs and identified that hepatocyte-targeting was related to ApoE adsorption on mRNA-LNP surface.

ApoE is a small, secreted protein that plays a crucial role in cellular lipid metabolism and endogenous cholesterol transport. It can bind to LDLR, which is abundant on hepatocyte surface, making it an essential component of endogenous targeting to hepatocytes. The ApoE-mediated endogenous targeting occurs through CME. Besides MC3-LNPs, 5A2-SC8-LNPs mentioned above, cKK-E12-LNPs also utilize ApoE-based hepatocyte-endogenous targeting. Paunovska et al. [91] observed that the ability of cKK-E12-LNPs to deliver mRNA to hepatocytes in ApoE-/- and LDLR-/- mice was significantly lower than in wild-type mice, confirming the ApoE-dependent targeting of cKK-E12-LNPs to hepatocytes. Comparing cKK-E12-LNPs and cKK-E15-LNPs having the same ionizable lipid core but different hydrophobic tail lengths revealed that cKK-E12-LNPs exhibited efficient mRNA delivery to hepatocytes in wild-type mice. These results indicate that changes in the structure of ionizable lipids in LNPs can affect ApoE-based endogenous targeting. Besides ionizable lipids, phospholipids can also affect ApoE adsorption on the mRNA-LNPs. LNPs with DOPE preferentially accumulate in the liver, and the interaction between LNPs with DOPE and ApoE was stronger than that with DSPC [118]. Protein coronas other than ApoE can be formed by modifying the lipid chemistry of LNPs. As a result, LNPs can enter hepatocytes through other endocytosis routes besides CME. Miao et al. [107] demonstrated that by incorporating alkyne and ester groups into cKK-E12-LNPs, serum albumin gets attached LNP surface instead of ApoE surface, and LNPs entered hepatocytes through CvME and micropinocytosis.

For liver diseases, mRNA-LNPs can be delivered to hepatocytes by endogenous targeting. In a mouse model of liver fibrosis and cirrhosis, Yang et al. [14] found that the ApoE-mediated endogenous targeting was not significantly affected. They synthesized LNPs containing ALC-0315, DSCP, cholesterol, and ALC-0159, and the LNPs successfully delivered HNF4A mRNA to hepatocytes through ApoE action. However, under specific pathological conditions, such as homozygous familial hypercholesterolemia (HOFH), the LDLRs almost disappeared, seriously affecting the ApoE- based endogenous targeting [119]. Therefore, active targeting strategies based on receptor-ligand binding are equally important in hepatocyte targeting, besides passive and endogenous targeting.

The asialoglycoprotein receptor (ASGPR) is present in many hepatocyte membranes. N-acetylgalactosamine (GalNAc) can be conjugated with mRNA-LNPs to actively target hepatocytes by ASGPR binding [120]. Notably, the hepatic uptake process dynamics of LNPs differs between the ApoE-LDLR-mediated endogenous targeting pathway and the GalNAc-mediated active targeting pathway [92]. Specifically, in the endogenous targeting pathway, LNPs rapidly enter the Disse space from the blood and gradually accumulate, then being slowly absorbed by the cells. In contrast, in the active targeting pathway, LNPs exhibit slower and more sustained cellular uptake, remaining in the blood for a longer period. Although active targeting can enhance the specificity of the mRNA-LNPs by incorporating additional targeting ligands, its implementation for hepatocyte targeting is less common due to increased formulation complexity. Currently, liver-specific delivery research predominantly employs passive and endogenous targeting.

Besides hepatocytes, other cells also play important roles in liver diseases and can serve as the target cells of mRNA NPs [121]. For example, HSCs are important effector cells in liver fibrosis [122], while liver sinusoidal endothelial cells (LSECs) are significant in immunotherapy and a proposed target for immunomodulation [113, 123].

However, HSCs are difficult to transfect, and the complexity of the liver microenvironment during liver fibrosis further compounds the difficulty of targeting HSCs. To address these challenges, Younis et al. designed a ligand-free mRNA-LNP containing CL15A6 lipid of hydrophobic scaffold structure (Fig. 7B) [108]. This formulation achieved successful transfection of over 80% of HSCs in vivo in liver fibrosis mice. The CL15A6-LNP had a diameter of approximately 80 nm, allowing it to pass through the fenestrated liver sinusoidal endothelium. Additionally, the LNP pKa was about 7.25, which made LNPs unrecognizable by ApoE, thus avoiding substantial interactions with hepatocytes. Further, CL15A6-LNP uptake by HSCs occurs through platelet-derived growth factor receptor β (PDGFRβ)-mediated CME. This process is associated with elevated serum PDGF levels during liver fibrosis and overexpression of PDGFRβ in HSCs. The reports on receptor-ligand active targeting strategy in mRNA delivery are nevertheless scarce because of the small proportion of HSCs in the liver. Mitchell et al. studied the active targeting strategy of mRNA delivery based on the neutral anisamide, a high-affinity ligand for the sigma receptor highly expressed in activated HSCs [112]. The anisamide-LNPs actively target HSCs, demonstrating potential in liver fibrosis treatment.

NPs larger than 150 nm can interact with LSECs effectively due to the fenestration size of hepatic vascular endothelium. Sato et al. [124] found that increasing the size of LNPs to 200 nm and raising the pKa to around 7.1 improve the targeting efficiency of LSECs by LNPs. Moreover, stabilin and mannose receptors were highly expressed on the LSEC membrane [125]. Roy et al. [109] demonstrated that mRNA-LNPs with a negative surface charge, achieved by replacing DSPC with distearoyl phosphatidylglycerol (DSPG), could be specifically absorbed by LSECs with the participation of stabilin receptors. Kim et al. [16] suggested that mannose-modified mRNA-LNPs could actively target LSECs and that increased PEG-lipid content (3%) in the formula could inhibit ApoE-mediated endogenous targeting. Xu et al. [113] employed mannosylated mRNA-LNPs to target LSECs and modulate the immune response in mice, successfully suppressing their allergic reactions to a peanut allergen, offering a promising intervention therapeutic approach to allergic diseases (Fig. 7C).

Interestingly, Dahlman et al. [110, 126] noted that altering the cholesterol structure of LNPs could regulate the ability of mRNA-LNPs to target organs and cells. LNPs with esterified or oxidized cholesterol can deliver mRNA to the liver microenvironment through lipoproteins, including LDL and very-low-density lipoprotein (VLDL) [126]. Further, higher mRNA expression could be selectively induced in hepatocytes, LSECs, and Kupffer cells by rationally changing the structure of oxidized cholesterol [110].

#### Other nanodelivery systems

Besides LNPs, various nanomaterials have been developed for mRNA targeted delivery to liver cells, with the majority being hybrid NPs. Prieve et al. [111] designed a PLN that targets the ASGPR by combining a GalNAc-targeted polymer micelle with an inert LNP. The NPs were designed to deliver mRNA encoding human ornithine transcarbamylase to hepatocytes, which could restore the levels of plasma ammonia and urinary orotic acid and prolong mice survival.

PLNs modified with the targeting peptide CTCE have also demonstrated potential in hepatocellular carcinoma (HCC) therapy. Xiao et al. [127] developed CTCE-modified PLNs to deliver p53 mRNA to HCC cells. These NPs specifically target the Cys-X-Cys (CXC) motif chemokine receptor 4 (CXCR4) that is highly expressed in these cells and release mRNA to play an anti-cancer role. Moreover, the combination of these hybrid NPs and anti-PD-1 (Programmed cell death protein 1) therapy displayed stronger anti-tumor effects, providing a promising therapeutic strategy for targeted treatment of liver cancer.

Similarly, Singh et al. [128] developed Selenium-based lactobionic acid-PEG-chitosan-SeNPs-mRNA nanocomplexes. The nanocomplexes actively targeted the ASGPR on HepG2 cell surface by LA ligand modification, which also demonstrated a favorable anticancer effect in liver cancer.

A new organic/inorganic hybrid mRNA NP based on biomimetic systems has been reported [129]. This system comprised virus-like mesoporous silica (V-SiO2), cationic polymer PEI, mRNA, and a lipid bilayer (LB). m@V-SiO2-P/LB NPs accurately targeted hepatocytes and exhibited much greater efficacy than did traditional mRNA-LNPs. Such biomimetic NP systems may serve as a basis for highly efficient mRNA targeted therapies.

### Lungs

**Fig. 8:**
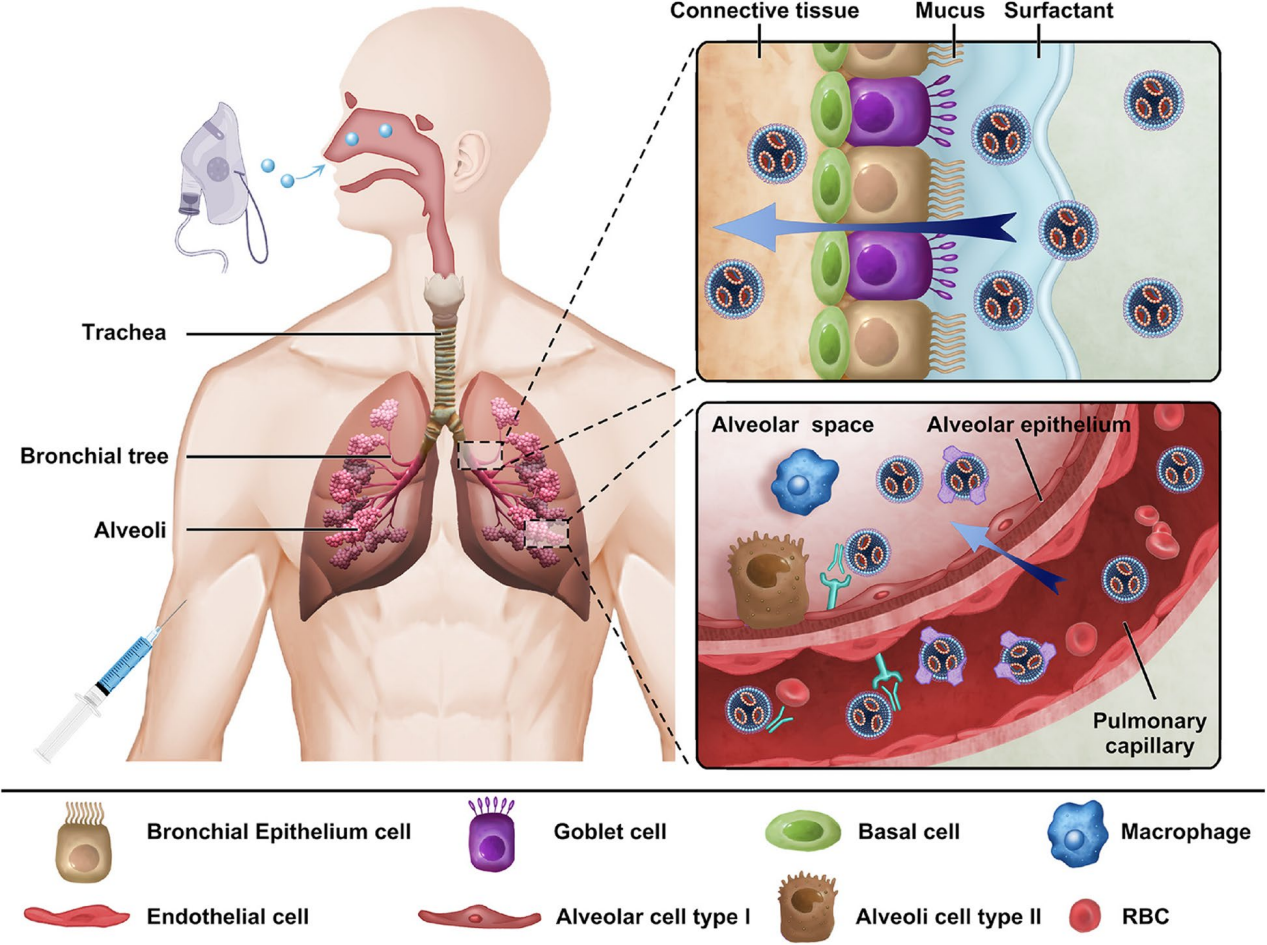
Schematic illustration of physiological barriers in the lung, and targeted delivery of mRNA NPs in the lung after intranasal or intravenous administration

The lungs are the main organ for gas exchange in the human body. The structure of the lungs is unique compared with that of other organs. The main functional areas of the lungs are the bronchus and alveoli. Alveoli, the terminal part of the bronchial tree, are surrounded by many capillaries. Alveoli are composed of a single layer of epithelial cells and rich in immune cells (especially macrophages). Because of the special structures and functions, there are two different routes to deliver mRNA NPs to the lungs: systemic administration (intravenous injection) and intranasal administration (Fig. 8).

Intravenous administration is one of the most common methods for pulmonary drug delivery. It can deliver large doses of mRNA NPs into the body in a short time and increase drug concentration in lungs to therapeutic level. Pulmonary endothelial cells, which are the main target cells in lung via intravenous administration, are continuous monolayers forming the surface of the pulmonary capillary lumen [130]. Due to nil fenestration in pulmonary capillaries, it is difficult for NPs to extravasate to alveolar epithelium through blood vessels [29]. Therefore, the ability of mRNA targeted delivery to other cell types besides pulmonary endothelial cells in alveoli by intravenous administration is limited. Moreover, resistance to liver aggregation is also a key concern in the study of intravenous administration to the lungs.

mRNA NPs can directly reach the alveoli through inhalation, avoiding non-specific uptake by other organs. This approach offers advantages such as safety, non-invasiveness, and high patient treatment adherence [131]. Nevertheless, there are certain barriers hindering the delivery efficacy of intranasally administrated NPs. Specifically, there are three main physiological barriers for mRNA NPs to reach epithelial cells in alveoli from the airway: mucus layers, pulmonary surfactant, and immune cells [132].

Mucus layers, covering the surface of the tracheas and bronchi, are the main barriers. Mucus layers are mainly mesh structures formed by proteins of the mucin family [133]. The pore size of the mucus layers is approximately 150 nm, and the layers are negatively charged [134]. Given the above characteristics, the size and charge of mRNA NPs affect their ability to pass through mucus layers. For example, if NPs are positively charged, electrostatic interaction will occur between NPs and mucus layers, resulting in NP retention [135]. Pulmonary surfactant (PS) contains a high proportion of lipids and a small amount of surfactant protein [136], which will form biomolecular coronas around mRNA NPs. Among these, surfactant proteins increase the uptake of NPs by alveolar macrophages [137], while interactions with surfactant lipids can control the overall uptake of NPs and counterbalance protein-mediated effects [138]. Immune cells such as macrophages are widely distributed in the respiratory tract and lungs, playing an innate defense function, particularly in the highly developed local immune system of the deep lung [139]. NPs with diameters larger than 240 nm are prone to engulfment by pulmonary macrophages [140], causing an unnecessary immune response. To overcome these barriers, various mRNA NPs have been developed; details are elaborated subsequently.

Thus, the administration route is a vital factor affecting the targeted delivery of mRNA NPs to the lungs, a special organ. Based on the administration routes and physiological barriers of the lungs, the targeting design strategies of mRNA NPs can differ. Next, we will introduce the design strategies of different NPs targeting the lungs in different administration routes and pulmonary milieu (Table 2).

**Table 2:** Summary of lung-targeted LNP-based mRNA delivery strategies

Delivery systems	Formulation (mol%)	Administration Route	Targeting features	Properties of mRNA NPs			Target cells	Ref.
				Size (nm)	Zeta (mV)	pKa		
***Passive targeting***								
LNP	5A2-SC8: DOPE: Chol: DMG-PEG: DOTAP 11.9: 11.9: 23.8: 2.4: 50	i.v.ss	50% permanently cationic lipids DOTAP pKa Vitronectin	113.1	-0.52	>11.0	Endothelial cells (<65%) Epithelial cells (<40%) Immune cells (<21%)	[106]
LNP	306O_10_: DOTAP: Chol: C_14_-PEG_2000_ 35: 40: 22.58: 2.5	i.v.	40% DOTAPs	~100	-3.5	/	Cells in blood vessels	[141]
LNP	cKK-E12: 18:0 DDAB: Chol: C_14_-PEG_2000_ 35: 44.5: 18: 2.5	i.v.	Cationic helper lipid 18:0 DDAB	106	1.81	7.7	Lung endothelial cells	[142]
LNP	306-N16B: DOPC: Chol: DMG-PEG_2000_ 50: 10: 38.5: 1.5	i.v.	An amide bond in the tail	81	-5.0	/	Endothelial cells (69.6%) Macrophages (18.9%) Epithelium cells (7.3%)	[143]
LNP	7C1: DOTAP: Chol: C_14_-PEG_2000_ 35: 5: 5: 55	i.n.	Cationic helper lipids DOTAP High molar ratio of PEG	65.5	/	/	Epithelial cell	[13]
LNP	DOTAP: MC3: DPPC: Chol: DSPE-PEG 40: 25: 10: 23.5: 1.5	i.n.	DSPE-PEG	38.8 ± 1.3	7.4 ± 1.4	/	Alveolar epithelial type 2 cells Fibroblasts	[144]
LNP	MC3: DSPC: Chol: DMG-PEG_2000_ 50: 10: 38.5: 1.5	i.n.	PEG shells	104.2 ± 30.5	/	/	Airway epithelial cells in cystic fibrosis	[145]
LNP	ionizable lipids: helper lipids: Chol: DMG-PEG_2000_ 50: 10: 38.5: 1.5	i.n.	Helper lipids DOPC or ESM	68.95 (DOPC) 72.31 (ESM)	Neutral charge	/	Epithelium cells	[146]
PLN	PEG-grafted PEI (PEG: 0.5%)	i.v.	PEG with amino or amino acid terminal groups PEG grafting ratio: 0.5%	~60	~10	/	/	[147]
PLN	DD90-C12-122: PEG-lipid 93:7	i.v.	Cationic polymeric NPs, DD90-C12-122	159.5±36.3	16	/	/	[148]
PLN	PBAE: DOPE: C_18_-PEG_2000_ 75:20:5	i.v.	PBAE Size	~100	/	/	Lung Endothelium Cells Lung Immune Cells	[149]
PLN	(DOTAP+PBAE): DOPE: Chol: DMG-PEG 50: 10: 38.5: 1.5	i.v.	Vitronectin Positive charge	100	~7		Lung endothelium Cells	[150]
PLN	I-DD3: DOPE: Chol: C_14_-PEG_2000_ 50: 25: 23.5: 1.5	i.v.	Ionizable amino-polyesters I-DD3	100 ± 10	10.0 ± 2.1	/	Lung endothelium Cells	[151]
PLN	AA03-DL-10: mDMG-PEG 9: 1	i.v.	The 5-carbon spacer in the backbone of the lipomer and the 4 carbons on the side chain Clathrin-mediated endocytosis	126	-22.6	/	/	[152]
***Active targeting***								
LNP	MC3: DSPC: Chol: PEG-lipid 50: 10: 38.5: 1.5 Conjugated with mAb specific for PECAM-1	i.v.	Active targeting (mAb specific for PECAM-1)	103.3 ± 0.2	-4.12 ± 0.1	/	Lung endothelial cells	[153]
sLNP	MC3: DSPC: Chol: DMG-PEG: DSPE-PEG 50: 10: 38: 1.75: 0.25 Conjugated with αPV1 antibody	i.v.	Active targeting (αPV1 antibody)	177	-6.34	/	Lung endothelial cells	[154]

#### LNPs

##### Systemic administration

LNPs are an ideal delivery system for mRNA, whether administered intranasally or intravenously. A significant observation on mRNA-LNPs passively delivered to the lungs by intravenous administration is that the addition of cationic lipids alone can redirect mRNA-LNPs from the liver to the lungs. This finding is noteworthy as it suggests a feasible strategy for pulmonary targeting while decreasing off-target delivery to the liver.

Using the SORT strategy, Siegwart et al. [106, 116, 155] added 50% permanently cationic lipids containing quaternary amino groups, such as DOTAP, Dimethyldidodecylammonium bromide (DDAB), and EPC, into the traditional mRNA-LNP formulation that was initially intended for liver targeting. This modification enabled the redirection of LNPs towards pulmonary delivery. The addition of cationic lipids significantly elevated the pKa of mRNA-LNPs to nine, far exceeding pKa of liver-targeted mRNA-LNPs (six - seven). This modification successfully enabled the selective transfection of approximately 40% of lung epithelial cells, 65% of pulmonary endothelial cells, and 20% of pulmonary immune cells. LoPresti et al. [141] observed that replacing the standard helper lipids in LNPs with cationic lipid DOTAP similarly leads to an enhancement of lung tropism for mRNA-LNPs. The available evidence indicates that the pulmonary targeting outcomes achieved by adding cationic lipids and high pKa values are closely related to the changes in the surface protein coronas of LNPs. LNPs modified with quaternary ammonium groups [106, 116, 156] with high pKa values, lead to a change in the adsorbed protein corona from ApoE to vitronectin. This altered protein corona preferentially binds to the αVβ3 integrin receptor, which is highly expressed in pulmonary microvascular endothelial cells, thereby making LNPs more prone to accumulate in the lungs [116].

A recent study has shown that incorporating cationic lipids as helper lipids in LNPs enhances their targeting specificity towards pulmonary endothelial cells [142]. Once endothelial cells are saturated, the LNPs target other cell types such as pulmonary immune and lung epithelial cells. Additionally, this study proposes an interesting hypothesis that the pre-treatment of cells may affect the in vivo distribution of mRNA-LNPs. Researchers have discovered that cells can alter their response to mRNA-LNPs by altering the processing of mRNA and subsequent protein production, such as translation initiation, complex formation, ribosome scanning, and initiation codon recognition. However, this hypothesis requires further in vivo experimentation for validation.

**Fig. 9:**
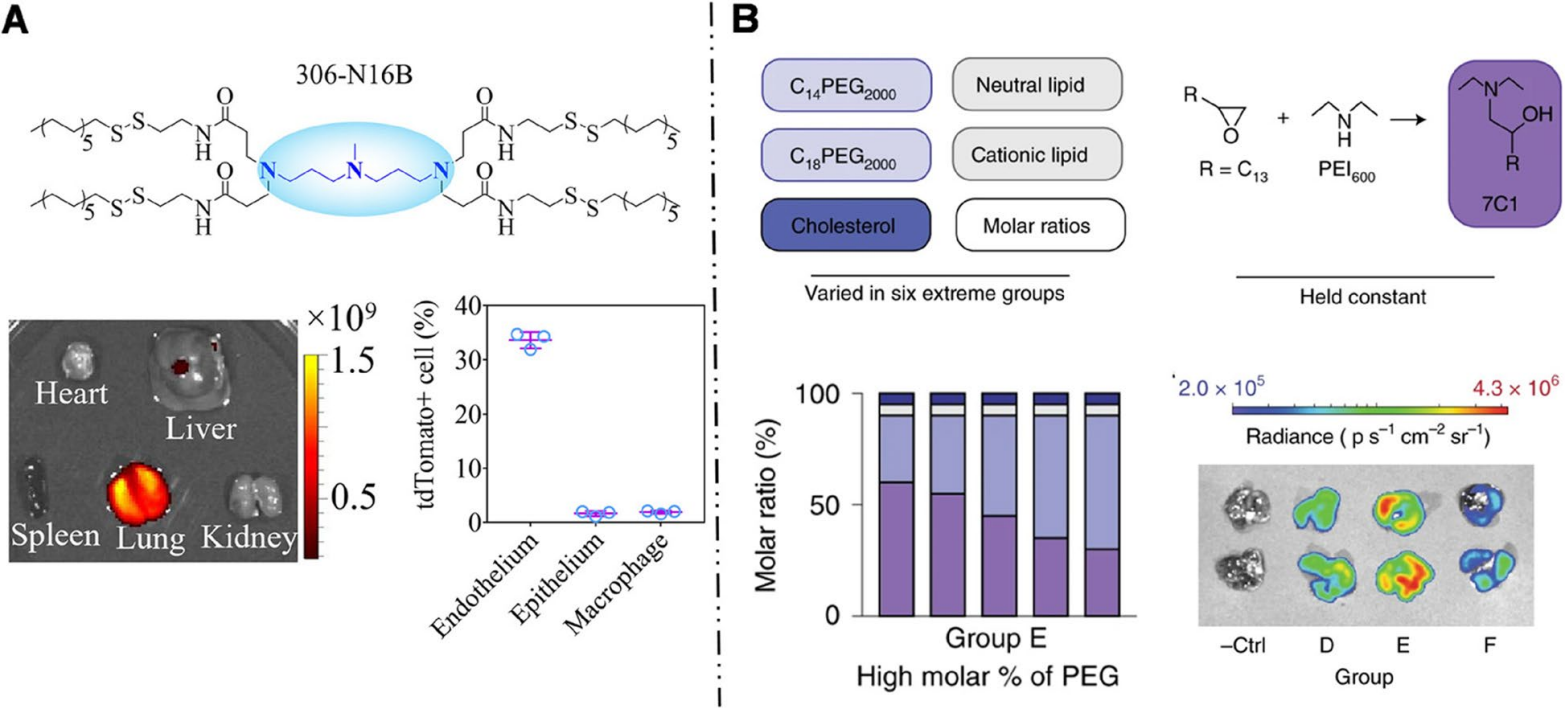
** A** 306-N16B (N-series) LNPs with an amide bond specifically delivered mRNA to lung via intravenous injection with high accuracy and pulmonary endothelium was mainly transfected. Reproduced with permission [143]. Copyright 2022, The Proceedings of the National Academy of Sciences. **B** A high proportion of PEG can promote the delivery of mRNA-LNPs to lung (group E) via nebulization. Reproduced with permission [13]. Copyright 2021, Springer Nature

Similarly, Qiu et al. [143] found that through the modification of the ionizable cationic lipid tail structure, specifically by introducing an amide bond (N-series LNPs), the delivery specificity of mRNA-LNPs can be accurately tuned to target the lungs with a high degree of precision. Among them, 306N16B LNPs exhibited the highest targeting ability. Their protein coronas mainly consisted of serum albumin, fibrinogen β chain, and fibrinogen γ chain. Based on these findings, fibrinogen was speculated to play an important role in directing pulmonary endothelial cell targeting, Previous studies have shown that fibrinogen coating can improve endothelial cell adhesion and endothelialization [157], which is consistent with the findings of Qiu’s study. However, besides fibrinogen, the potential involvement of other proteins remained unclear, and further investigation is required to determine whether a single protein or multiple proteins act in concert. Additionally, modifying the head structure of N-series LNPs allows for selective targeting of various types of lung cells including pulmonary endothelial cells, macrophages, and epithelial cells (Fig. 9A) [143].

Overall, in the absence of active ligands, the addition of cationic lipids and structural variations in cationic lipids can both impact protein coronas adsorbed onto the LNP surface, thus affecting their organ-level and cellular-level targeting. These findings emphasize the potential of cationic lipids for lung-specific mRNA delivery.

Currently, limited attention is given to the active targeting strategy of adding targeting molecules to LNPs, possibly due to the relatively underdeveloped research on ligands for targeting lung cells. Based on our investigation, currently only two ligands have been utilized for pulmonary active targeting strategies for mRNA-LNPs. Specifically, pulmonary epithelial cells can internalize plasma membrane vesicle-associated protein 1 (PV1) through CvME [158]. Building upon this, Li et al. [154] successfully enhanced mRNA expression in lung epithelial cells by covalently binding αPV1 antibodies to the surface of mRNA-LNPs. Besides, platelet endothelial cell adhesion molecule (PECAM) serves as a target for endothelial cells [159]. Therefore, Parhiz et al. [153] coupled monoclonal antibodies targeting PECAM1 with mRNA-LNPs and successfully targeted pulmonary endothelial cells. As a result, the reported protein signal in the lungs increased 25-fold compared to that in non-targeted counterparts. The study further revealed that the pulmonary delivery of PECAM-1 targeted mRNA-LNPs was independent of ApoE.

##### Intranasal administration

The first obstacle for LNPs in the respiratory tract is the mucous layer on the tracheal surface. The negative charge of the mucus hinders LNP penetration through the mucus layers. Positively charged LNPs are trapped in the mucus layer due to electrostatic interactions. Hydrophilic NPs with neutral or negative charges can penetrate the mucus layers by diffusion effects, with neutral NPs diffusing more rapidly than negatively charged ones [160]. However, charge is not the only factor determining the ability of NPs to pass through the mucus layers; size is also an important factor. Small NPs can effectively move through the mucus layer, while the movement of larger NPs is significantly hindered. Bertrand et al. [161] proposed that NPs smaller than 100 nm can more effectively penetrate the mucus layer than those larger than 250 nm for effective drug release. Furthermore, PEGylation of mRNA-LNPs can improve the stability of LNPs during nebulization, making it an effective strategy to facilitate mucus penetration [162]. A high proportion of PEG density can limit LNP aggregation and promote cellular uptake. Lokugamage et al. [13] designed LNPs with a 55% PEG-lipids and 5% cationic helper lipids (DOTAP) composition to deliver the mRNA encoding neutralizing antibodies to the lungs via nebulization, resulting in successful protection against the influenza A virus in mice (Fig. 9B).

Besides, during diseased lungs, PEGylation is crucial for pulmonary targeted delivery of mRNA-LNPs. In a pulmonary fibrosis mouse model, neutral surface-charged mRNA-LNPs, facilitated by PEG-lipids, transfected both alveolar epithelial cells and lung fibroblasts via intranasal administration [144]. Likewise, in hereditary pulmonary diseases, including cystic fibrosis (CF) and primary ciliary dyskinesia (PCD) [145, 163], mRNA-LNPs encoding genetic information were injected into PEG shells and delivered via intranasal administration, successfully targeting airway epithelial cells in the corresponding disease mouse models, with the proteins expressed only in the lungs.

Besides the “stealth” properties of PEG-lipids, helper lipids play a crucial role in stabilizing mRNA-LNPs via intranasal administration and facilitating their penetration through the mucus layer. For instance, Tam et al. selected four phosphatidylcholine (PC) lipids as helper lipids in LNPs, among which 1, 2-dioleoyl-sn‑glycero-3-phosphocholine (DOPC) and egg sphingomyelin (ESM) can better stabilize mRNA-LNPs in the airway, enabling them to penetrate the mucus layer more effectively. mRNA-luciferase activity was detected in the lungs even 24 h after administration, indicating the stability and efficacy of these lipids [146].

#### Polymers/ hybrid polymers

##### Systemic administration

Like the LNPs, cationic polymers are widely used in targeted pulmonary delivery strategies. Commonly used cationic polymers include PEI [147, 164], PBAEs [148–150, 165], and polyester materials [151, 166–168]. PEI is widely used as a cationic polymer carrier for nucleic acid delivery [60, 169]. However, excessive positive charge and non-degradability negatively affect its biocompatibility and stability [170]. Toxicity of PEI can be reduced through PEGylation when administered systemically [171]. Huang et al. [164] discovered that PEGylated PEI (PEG-PEI) systems had higher colloidal stability and lower toxicity than PEI alone. In-depth study has shown that various PEG terminal groups and grafting ratios can affect the biodistribution and cellular uptake of PEG-PEI [172]. Ke et al. [147] reported that mRNA-PEG-PEI (with a PEG grafting ratio 0.5%), having an amino-terminal group and amino acid residue, expressed the highest in the lung, with mRNA mainly transfected into pulmonary immune cells.

**Fig. 10:**
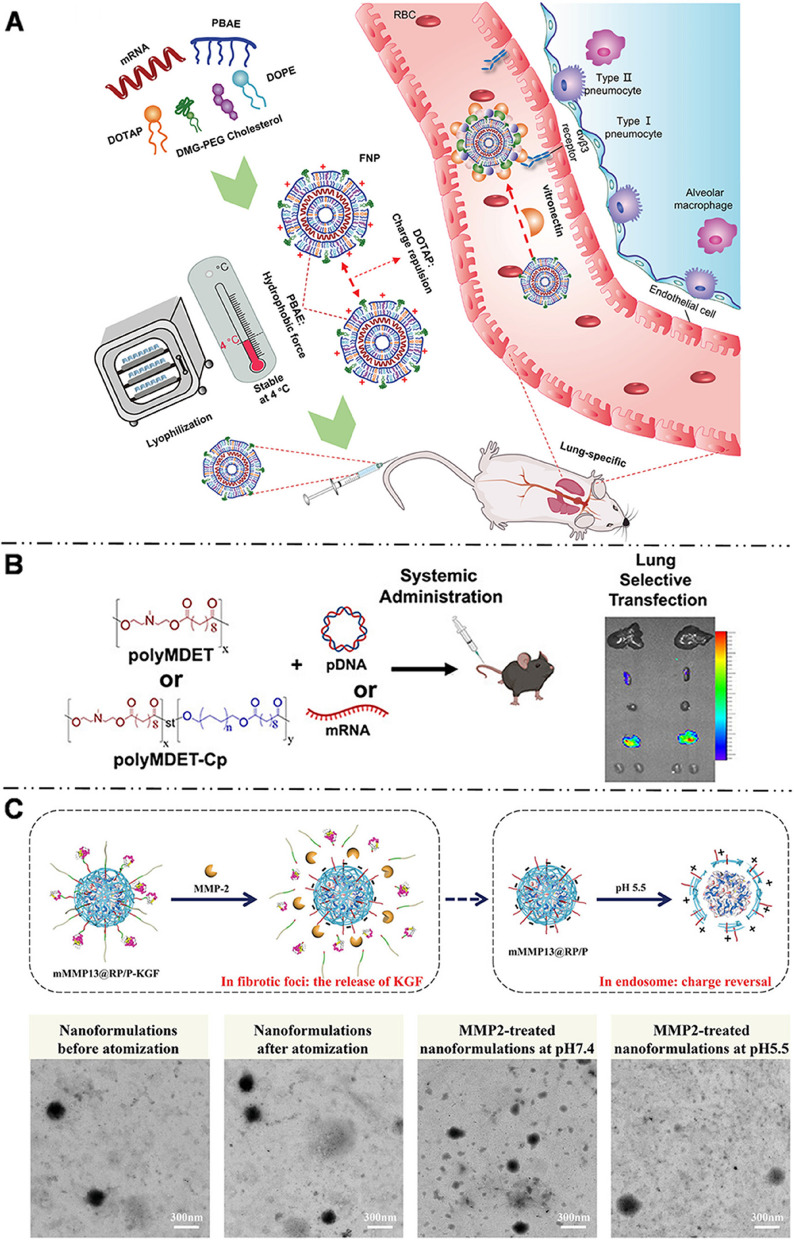
** A** Schematic illustration of unique characteristics of five-element nanoparticles (FNPs) for Lung-specific mRNA delivery. Reproduced with permission [150]. Copyright 2022, American Chemical Society. **B** Schematic illustration of the ionizable polyester nanoparticles (polyMDET or polyMDET-Cp) for lung-selective nucleic acid transfection. Reproduced with permission [168]. Copyright 2022, American Chemical Society. **C** Stimulus-responsive bifunctional peptide hybrid NPs (mMMP13@RP/P-KGF) used for the treatment of idiopathic pulmonary fibrosis, and illustration and TEM images of its MMP2-responsive and pH-sensitive abilities. Reproduced with permission [174]. Copyright 2022, John Wiley and Sons

PBAEs, as an alternative carrier of PEI, stand out in the design of lung-targeted cationic polymers because of their simpler synthesis, excellent biodegradability, and good lysosomal escape ability [173]. Kaczmarek et al. [148] reported for the first time in 2016 that PBAEs-PEG delivered mRNA specifically to the lungs through intravenous injection. Subsequently, they adjusted the polymer structure and formulation (PBAEs based on diacrylate-amine backbone incorporating the alkylamine and end cap amine), and the optimized PBAEs-PEG specifically delivered mRNA to pulmonary endothelial cells and pulmonary immune cells, exhibiting twice the efficacy of basic NPs [149]. The same PBAEs-PEG formula had different efficiencies when delivering different nucleic acids. In lung targeting research, the ability to deliver mRNA was two orders of magnitude higher than for that to deliver pDNA [165]. Cao et al. [150] designed five-element NPs (FNPs) composed of PBAEs as auxiliary polymers, DOTAP, DOPE, cholesterol, and DMG-PEG, which exhibited a high specificity of pulmonary targeting (Fig. 10A). During proteomic analysis, FNPs were found to follow the SORT principle, and their pulmonary endogenous targeting mechanism was the adsorption of vitronectin as the protein coronas on mRNA NP surface and interaction with αvβ3 integrin receptors expressed on pulmonary capillary endothelial cells.

Polyester NPs (PNPs) are also interesting candidates for mRNA delivery due to their excellent biocompatibility and biodegradability. The chemical characteristics of PNPs are crucial in determining their organ selectivity. Kowalski et al. [151] synthesized new ionizable amino-polyesters (APEs) composed of lactones and tertiary amino-alcohols in one step using ring-opening polymerization. The APEs were further combined with LNPs containing PEG, cholesterol, and DOPE components. Eight groups of APEs-LNPs were synthesized based on different chemical structures of lactone and amino alcohols. Among them, I-DD3 with alkyl side chains in lactone and four tertiary amine groups in amino alcohol achieved optimized pulmonary localization of mRNA and was primarily found to transfect pulmonary endothelial cells. Changing the structure of amino alcohols alone altered the targeting organ of mRNA delivery. Additionally, the alkyl chain in polyester also influenced the organ-targeting ability of mRNA. The Siegwart lab proposed a pulmonary-targeted functional polyester-based mRNA-vehicle class with tunable molecular weight. By changing the length and molar ratio of the alkyl chain in polyester, the delivery selectivity between the lungs and the spleen can be tunable [166, 167].

Interestingly, the pKa of hydrophobic ionizable polyester targeting pulmonary immune cells was found to be less than five, contrary to the result of pKa > 9 observed in LNPs (Fig. 10B) [168]. It could be relevant to the difference in intracellular endocytosis pathways. The endocytosis pathway of pulmonary immune cells was mediated by CME and lipid raft. It might also be related to the difference in physicochemical properties or the composition of protein coronas on PNP surface. Similarly, Abd Elwakil et al. synthesized polyester lipomers based on ε-decalactone (ε-DL) monomers named AA03-DL-10 that can preferentially target the lungs. In vitro experiments have shown that AA03-DL-10 is taken up by cells via CME [152]. The 5-carbon spacer in the backbone of the lipids and the four carbons on the side chain were of utmost importance for efficient pulmonary targeting.

Similarly, cationic amphiphilic polyaspartamide derivatives with a side chain of diethylenetriamine (PASP (DET)) have also shown potential for delivering mRNA to the lungs. Yum et al. [175] synthesized PASP (DET/CHE) with a cyclohexyl ethyl (CHE) group, which caused significant mRNA expression in the lungs and even demonstrated mRNA transfection efficiency nearly 10 times higher than lipid materials commonly used on the market. Park et al. [176] found that PEG-modified PASP (DET) (with a PEG/polymer ratio of 1:1) can induce mRNA expression in the lungs, but caution should be exercised as high PEG content may decrease the efficiency of mRNA delivery after intravenous injection. mRNA transduction and translation in the lungs were completely absent when the PEG/polymer ratio was 10:1.

In the mouse model of lung diseases such as cystic fibrosis (CF), biodegradable chitosan-coated PLGA could deliver mRNA to the lungs by both intravenous administration and intranasal administration [177, 178]. In their study, Haque et al. [177], considering the enhancement of mucus clearance in the CF model, opted for intravenous administration as an alternative to inhalation delivery. They discovered that chitosan-coated PLGA NPs could similarly target the lungs effectively.

##### Intranasal administration

PBAEs can be employed not only for intravenous administration but also for intranasal delivery. For example, hyperbranched poly(beta amino esters) (hPBAEs) have been synthesized to deliver mRNA to the pulmonary epithelium by inhalation [179]. Moreover, adding the thiol component to the PBAEs enabled the co-delivery of short crRNAs and long mRNAs to the cells within the alveolar space [180]. Zhang et al. [174] specifically designed stimulus-responsive bifunctional peptide hybrid NPs, comprising a ribosomal protein-condensed mRNA core, bifunctional peptide-modified coronas, and a PEGylated shell with keratinocyte growth factor (KGF) as the key stimulus-responsive component. The NPs exerted effects by being inhaled into the alveoli, where they penetrated fibrotic foci and responded to matrix metalloproteinase 2 (MMP2), leading to the detachment of the outer KGFs and targeting cells with enriched integrins for mRNA delivery (Fig. 10C). These NPs were designed to treat idiopathic pulmonary fibrosis by clearing intrapulmonary extracellular matrix and re-epithelializing the alveolar epithelium.

### Immune organ and cells

#### Spleen

**Fig. 11:**
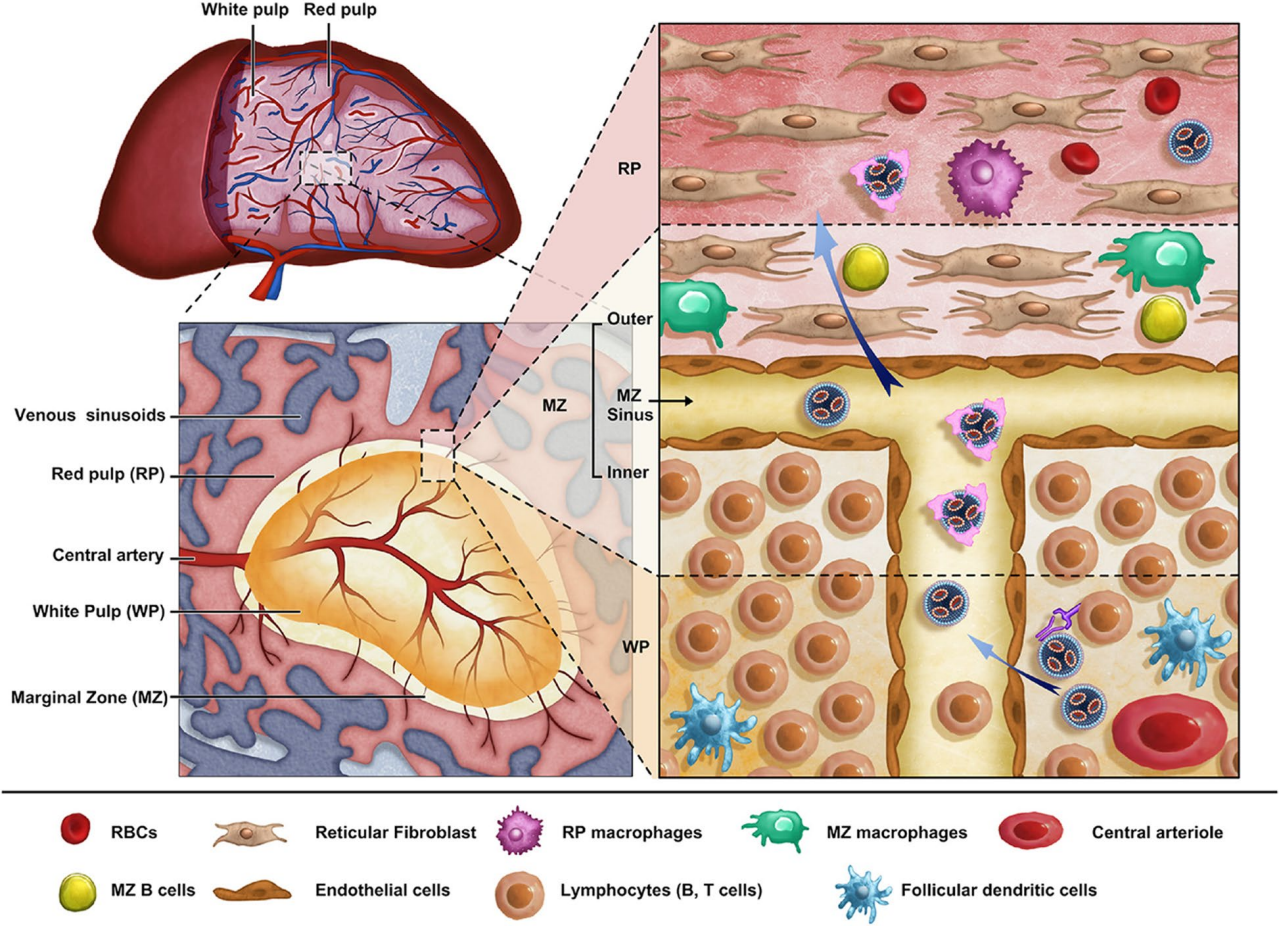
Schematic illustration of the microscopic anatomical structure of spleen and the journey of mRNA NPs in spleen via intravenous injection. The spleen is divided into three main areas: red pulp (RP), white pulp (WP), and marginal zone (MZ). RP is rich in macrophages, reticular cells and related reticular fibers, while the WP is involved in the proliferation of both B and T cells. The MZ comprises specialized macrophages

The spleen, one of the most important lymphoid organs for immune functions, contains various subsets of dendritic cells (DCs), T cells, B cells, and macrophages, and is an ideal target organ for immunization with mRNA NPs. There are three zones in spleen: red pulp (RP), white pulp (WP), and marginal zone (MZ) (Fig. 11) [181]. The RP is rich in macrophages, reticular cells, and related reticular fibers, while the WP is involved in the proliferation of both B and T cells. The MZ comprises specialized macrophages. The spleen has neither lymphatic vessels nor lymphatic sinuses but many blood sinuses [182]. Such a special microvascular environment and slow blood circulation make intravenous injection a usual administration route of spleen-targeted NPs. However, NP accumulation is only 15% of the injected dose in the spleen, and the liver absorbs more than 85% of the dose as blood flow to liver exceeds that to the spleen. Therefore, the liver is considered the main biological obstacle to spleen-targeted delivery [183]. Increased size of NPs can effectively reduce liver uptake and increase splenic capture. From the WP to the RP, NPs larger than 200 nm in diameter are easily captured and internalized by spleen macrophages [184]. Only NPs with a size of 100–200 nm can successfully escape from the clearance by macrophages, pass through MZ, and finally reach the B or T cell area [185]. Liver was also bypassed by modifying the pKa and zeta potential of NPs [41, 186]; this is detailed in the following paragraph (Table 3).

**Table 3:** Summary of spleen-targeted LNP-based mRNA delivery strategies

Delivery systems	Formulation (mol%)	Administration route	Targeting features	Properties of mRNA NPs			Target cells	Ref.
				Size (nm)	Zeta (mV)	pKa		
***Passive targeting***								
LNP	DODAP: DOPE: Chol: DMG-PEG 28.5: 60: 10: 1.5	i.v.	60% DOPE	125 ± 22	-10 ± 8.6	/	APCs in the spleen	[187]
LNP	10A1P16: MDOA: Chol: DMG-PEG_2000_ 25: 30: 30: 1	i.v.	Iphos chain lengths zwitterionic helper lipids	150	-6	6	Macrophages (30%) B cells (6%)	[155]
LNP	C12-200: DSPC: Chol: C14-PEG_1000_ 40: 10: 18.5: 1.5	i.v.	DSPC	167.2	/	/	/	[118]
LNP	5A2-SC8: DOPE: Chol: DMG-PEG: 18PA 16.7: 16.7: 33.3: 3.3: 30	i.v.	18PA pKa β2-GPI	142.1	-2.11	3.97	B cells (12%) T cells (10%) Macrophages (20%)	[106]
LNP	4A3-SC8: DOPE: Chol: DMG-PEG: BMP 15: 15: 30: 30: 3: 20	i.v.	Negatively charged lipids, BMP	~120	Neutral charge	/		[186]
LNP	SS-EC: DOPE: Chol: DSG-PEG_2000_ 59.5: 12.24: 26.54: 1.72	i.v.	DSG-PEG_2000_ Size Enrichment of immunoglobulins	83	3	/	APCs in the spleen	[188]
LNP	306O_10_: PS: Chol: C14-PEG_2000_ 35: 40: 22.58: 2.5	i.v.	40% PS	~110	~-6	5.5	Cells in WP (mostly B cells)	[141]
LNP	OF-Deg-Lin: DOPE: Chol: C14-PEG_2000_ 35: 16: 46.5: 2.5	i.v.	Ester bonds	75 ± 10	/	5.7	B cells in the spleen	[189]
PLN	Gardiquimod-loaded PLGA-core/lipid-shell hybrid NP	i.v.	Gardiquimod	~400	~20	/	DCs in the spleen	[190]
***Active targeting***								
LNP	MC3: DSPC: DSPE-PEG_2000_: DSPE-PEG_5000_-Mal: Chol 50: 10: 1.5: 0.5: 38 Conjugated with anti-CD3 antibody	i.v.	Active targeting (anti-CD3 antibody)	168.5	-6.2 ± 5	/	Splenic CD3e^+^ T cells	[191]
LNP	ALC-0307: PC: Chol: PEG 50: 10: 38.5: 1.5 Conjugated with anti-CD4 antibody	i.v.	Active targeting (Anti-CD4 antibody)	88.37 ± 4.2	/	/	T cells in the spleen	[192]

##### LNPs

LNPs are regarded as promising carriers for targeting extrahepatic organs, owing to their biocompatibility, customizable size, and surface functionalization. Adjusting helper phospholipids affect the ability of LNPs to target spleen [118, 187]. When the proportion of helper phospholipids DOPE in mRNA-LNPs was adjusted to 60%, luciferase activity was observed in the spleen, which far exceeded that in the liver and lung [187]. Siegwart’s team designed a class of multi-tailed ionizable phospholipids (iPhos) with varying alkyl chain lengths to formulate multicomponent LNPs (called iPLNPs) [155]. Their research discovered that elongating the alkyl chain length of iPLNPs shifted the mRNA-iPLNPs delivery from the liver to the spleen. Moreover, Zhang et al. [118] reported that without changing other components in LNPs, the replacement of DOPE with DSPC delivered more mRNA to the spleen, which may be related to antagonizing the DSPC-LNPs and ApoE interaction.

**Fig. 12:**
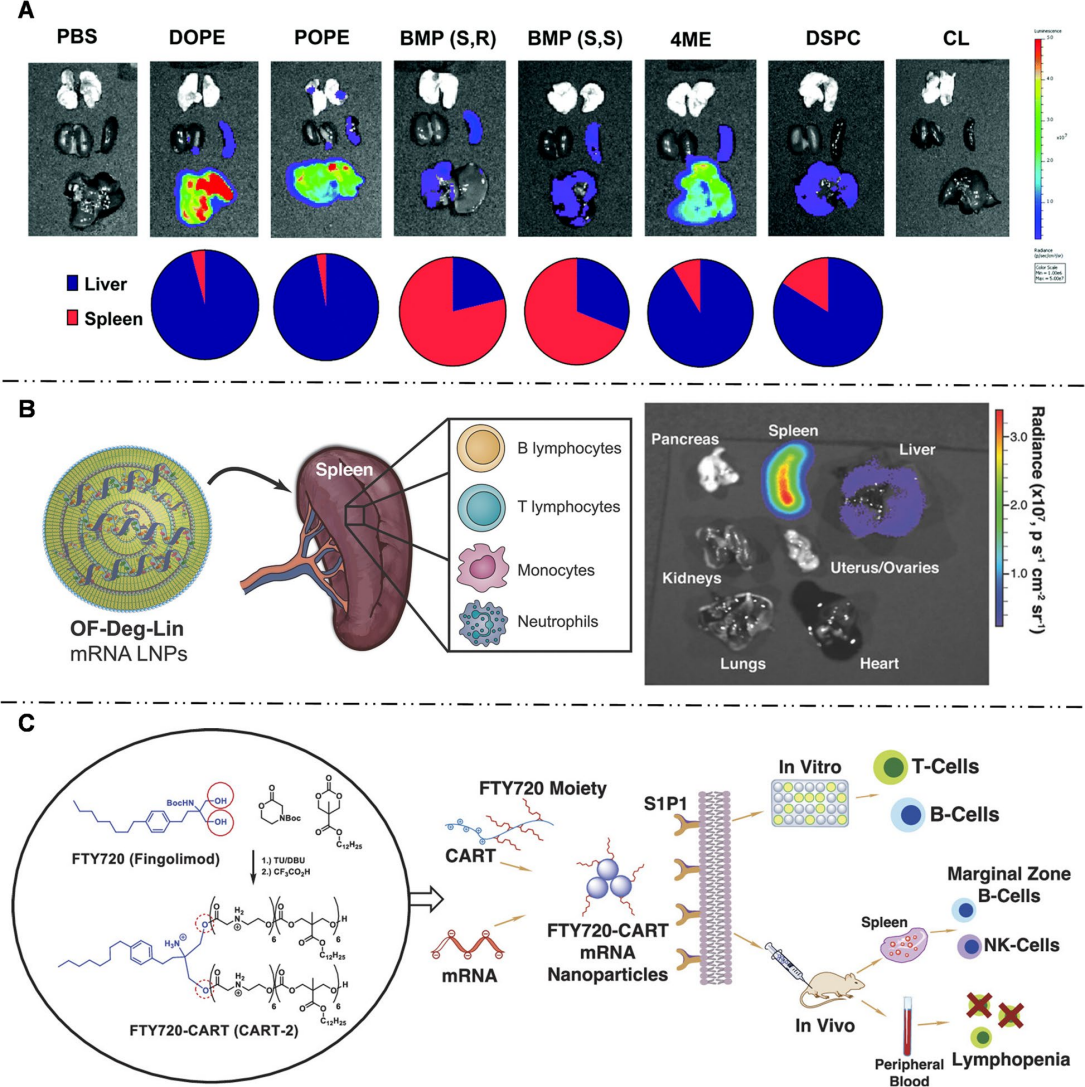
** A** Negatively charged phospholipids (such as BMP) aided spleen delivery of mRNA-LNPs. The fluorescein imaging of organs in vivo and liver to spleen ratio of luminescence were presented. Reproduced with permission [186]. Copyright 2022, The Royal Society of Chemistry. **B** Structures of ionizable lipids affected the targeted delivery of mRNA to the spleen. Functional luciferase expression results showed that biodistribution of OF-Deg-Lin FLuc mRNA LNPs was predominantly in the spleen. Reproduced with permission [189]. Copyright 2017, John Wiley and Sons. **C** Synthesis of fingolimod-conjugated CARTs and schematic illustration of targeted delivery of mRNA to lymphocytes in spleen. Reproduced with permission [195]. Copyright 2022, American Chemical Society

In detailed studies, down-modulation of pKa and zeta potentials on mRNA-LNP surface enhanced spleen tropism of LNPs. Following the SORT strategy, Siegwart et al. [106, 116] proposed five-component mRNA-LNPs formed by adding 10–40% anionic lipids (such as 18PA, 14PA, and 18BMP). As a result, the pKa value on mRNA-LNP surface was reduced to 2–6, while the neutral zeta potential remained constant, promoting spleen-specific delivery. Subsequently, replacing neutral phospholipids with anionic lipids also achieved the same splenic targeting effect (Fig. 12A) [186]. Incorporation of anionic lipids effectively reduces the pKa of LNPs, leading to the preferential adsorption of β2-glycoprotein I (β2-GPI) [193]. LNPs also facilitate mRNA delivery to the spleen via a β2-GPI-mediated endogenous targeting mechanism [116, 194]. However, a recent study assessed the changes of protein coronas on mRNA-LNP surface accumulated in spleen after repeated administration, and the result was the enrichment of immunoglobulins [188]. It suggests that immunoglobulins also play an important role in the protein coronas on spleen-targeting LNP surface. Whether the protein coronas act together or promote spleen targeting independently remains inconclusive. Negative charge on LNP surface increased the spleen targeting ability of LNPs. Kranz et al. [41] used DOTMA (cationic lipid) and DOPE (helper lipid) to form mRNA-Lipoprotein X (LpX)s. It was observed that mRNA-LpXs with a zeta potential around − 35 mV generated a signal exclusively in the spleen, with no significant expression observed in the liver. LoPresti et al. [141] used phosphatidylserine as helper lipids, maintaining a four-component LNP system. The surface of these mRNA-LNPs displayed a weak negative surface charge, thus reproducing the same spleen tropism.

Furthermore, the structures of ionizable lipids affect the protein expression of mRNA in the spleen. Fenton et al. developed an ionizable lipid OF-Deg-Lin containing biodegradable ester bonds (Fig. 12B) [189, 196]. Although the mRNA-LNPs composed of OF-Deg-Lin accumulated stronger in the liver than in the spleen, the mRNA expression in the spleen was over 85% while being hardly expressed in the liver. The reason might be that the ester bond degraded quickly in the liver before inducing protein expression in the hepatocytes (e.g., during cell internalization). Conversely, OF-Deg-Lin-LNPs might have been preserved in the spleen, retaining their ability to generate functional protein expression following uptake in the resident splenic cells. Although the mechanism of OF-Deg-Lin mRNA-LNPs promoting mRNA expression in the spleen is unknown, it is clear that the ester bond is crucial for delivering mRNA to the spleen [197].

At present, active spleen targeting mainly uses antibodies targeting antigens on lymphocyte surface, such as antiCD3-LNPs and antiCD4-LNPs, which results in mRNA-LNPs accumulating in the spleen leading to potential applications in immunotherapy [191, 192].

##### Polymers/Polymer-Lipids

Like that in LNPs, the incorporation of negatively charged components into polymers and reasonable variations in the polymer side chains can also deliver mRNA to the spleen. Cationic polymers can be converted into zwitterionic phosphatized polymers (ZPPs) by phospholipidation functionalization [198]. The pKa on the polymer surface was reduced from 8.0 to about 6.5 by negative phosphate group introduction on side chains, which promoted spleen tropism and mRNA expression. This shows that zwitterionic phospholipidation has great potential in transforming defective cationic polymers into effective mRNA NPs for spleen-targeted delivery.

Further, other copolymers have the ability to transfect cells in the spleen. Palmiero et al. [199] presented an ionizable poly (β-amino ester) co-poly (caprolactone) terpolymers modified by PEG-lipid able to deliver mRNA to the spleen preferentially. Furthermore, Jiang et al. [200, 201] found that poly(amine-co-ester) (PACE) polymers were effective in delivering mRNA to the spleen, regardless of the end groups. These results suggested that organ-targeting ability of PACE-based NPs was determined by polymer backbone. Although the stealth and stability of PEG may be useful in the targeted delivery to non-liver organs, no beneficial effects were observed for PACE-PEG in vivo distribution, while adding higher amounts of PEG counterintuitively reduced the expression of mRNA in the spleen [55]. This suggested that the usage scenarios and the amount of PEG added need to be carefully considered [202].

Moreover, PLNs have also been referenced in the context of spleen-targeted research. Yang et al. [190] designed PLNs composed of PLGA-core/lipid-shell to co-deliver mRNA and gardiquimod (adjuvant). The PLNs administered intravenously resulted in mRNA transfection efficiency in the spleen and a strong immune response in the B16-ovalbumin (OVA) melanoma tumor mouse model. The study showed that the simultaneous delivery of antigen and adjuvant through the core/shell NPs was beneficial in inhibiting tumor growth. These PLNs combine the advantages of both polymers and lipids, offering a diverse platform for potential therapeutic applications in spleen targeting.

New mRNA polymer-carriers targeting immune cells, dubbed charge-altering releasable transporters (CARTs) [195, 203–206], are gaining attention. Different from conventional cationic polymers, CARTs undergo dynamic changes of degradation and charge-neutralizing intramolecular rearrangement on cellular entry. In this process, CARTs degrade from cationic polymers to nontoxic small molecules, releasing mRNA rapidly in cells. The latest CART designs contained unsaturated lipid blocks of side chains or an oligo (serine ester) backbone [203–205]. The CARTs in vivo were mainly located in the spleen, which is related to a large number of lymphocytes in the spleen. Furthermore, CARTs can conjugate with fingolimod which can bind to the sphingosine-1-phosphate receptor 1 (high expression in lymphocytes) and deliver mRNA to MZ B cells and natural killer (NK) cells via active targeting (Fig. 12C) [195]. This provided an idea for mRNA NPs for active splenocyte targeting.

#### Immune cells

With the outbreak of the COVID-19 pandemic, research and application of mRNA vaccines have attracted extensive attention. Many encouraging mRNA vaccines are reported for infectious diseases and cancer [207]. Targeting specific immune cells (such as APCs and T cells) with mRNA NPs is an important research direction in mRNA vaccine development to enhance their immunogenicity. The focus of this section is to discuss the design strategies for mRNA NPs targeting APCs and T cells (Table 4).

**Table 4:** Summary of LNP-based mRNA delivery strategies targeting APCs and T cells

Delivery systems	Formulation (mol%)	Administration Route	Targeting features	Properties of mRNA NPs			Target cells	Ref.
				Size (nm)	Zeta (mV)	pKa		
***Passive targeting***								
LNP	YK009: DSPC: Chol:DMG-PEG_2000_ 50: 10: 38.5: 1.5	i.m.	Clathrin- and caveolae-mediated endocytosis Different ester bond position Two branched saturated tails with different lengths	93.2 ± 3.5	0.9 ± 0.6	6.512	DCs	[208]
LNP	PPZ-10: DOPE: Chol: C_18_PEG_2000_ 35: 16: 46.5: 2.5	i.v.	PPZ-A10 (piperazine-based lipids with shorter C10 carbon chains)	98.3	/	7.0	Kupffer cells(60%) Spleen macrophages(50%) Spleen DCs(30%) Liver DCs(20%)	[209]
LNP	MC3: DOPE: β-sitosterol: DMG-PEG_2000_ 50: 10: 38.5: 1.5	i.m.	DOPE and β-sitosterol	95 ± 5	-0.1 ± 3	/	DCs	[210]
LNP	C14-4: DOPE: Chol (7α-hydroxycholesterol): DMG-PEG_2000_ 35: 16: 46.5: 2.5	/	25% 7α- hydroxycholesterol	~100	~-8	~6.25	Primary human T cells	[211]
PLN	PLGA core Lipid layer (70% DOPE + 30% DOTMA)	/	70% DOPE and 30% DOTMA	~250	~-10	/	DCs	[212]
PLN	cationic polymer (SW-01) core PEG-lipid shell	i.m.	Size Lipid composition	~200	/	/	DCs at injection site DCs in spleens and lymph nodes	[213]
***Active targeting***								
LNP	DLin-DMA: DSPC: Chol (mannosylation): DMG-PEG_2000_ 40: 10: 48: 2	i.m. i.d.	Active targeting (Mannosylation) Disaccharide	151.1 ± 0.86	/	/	APCs	[214]
LNP	ALC-0315: PC: Chol: PEG-lipid 50: 10: 38.5: 1.5 Conjugated with anti-CD5 antibody	i.v.	Active targeting (anti-CD5 antibody)	80	/	/	T cells	[215]
LNP	PbAE/ PGA-anti-CD8	i.v.	Active targeting (anti-CD8 antibody)	106.9 ± 7.2	4±2	/	T cells	[216]
APN	MC3: DSPC: Chol: DMPE-PEG_2000_ 50: 10: 38.5: 1.5 Conjugated with pMHCI molecules	i.v.	Active targeting (pMHCI molecules)	107.9 ± 7.34	-22.73 ± 4.7	/	Ag-specific T cell subsets	[217]

##### APCs

**Fig. 13:**
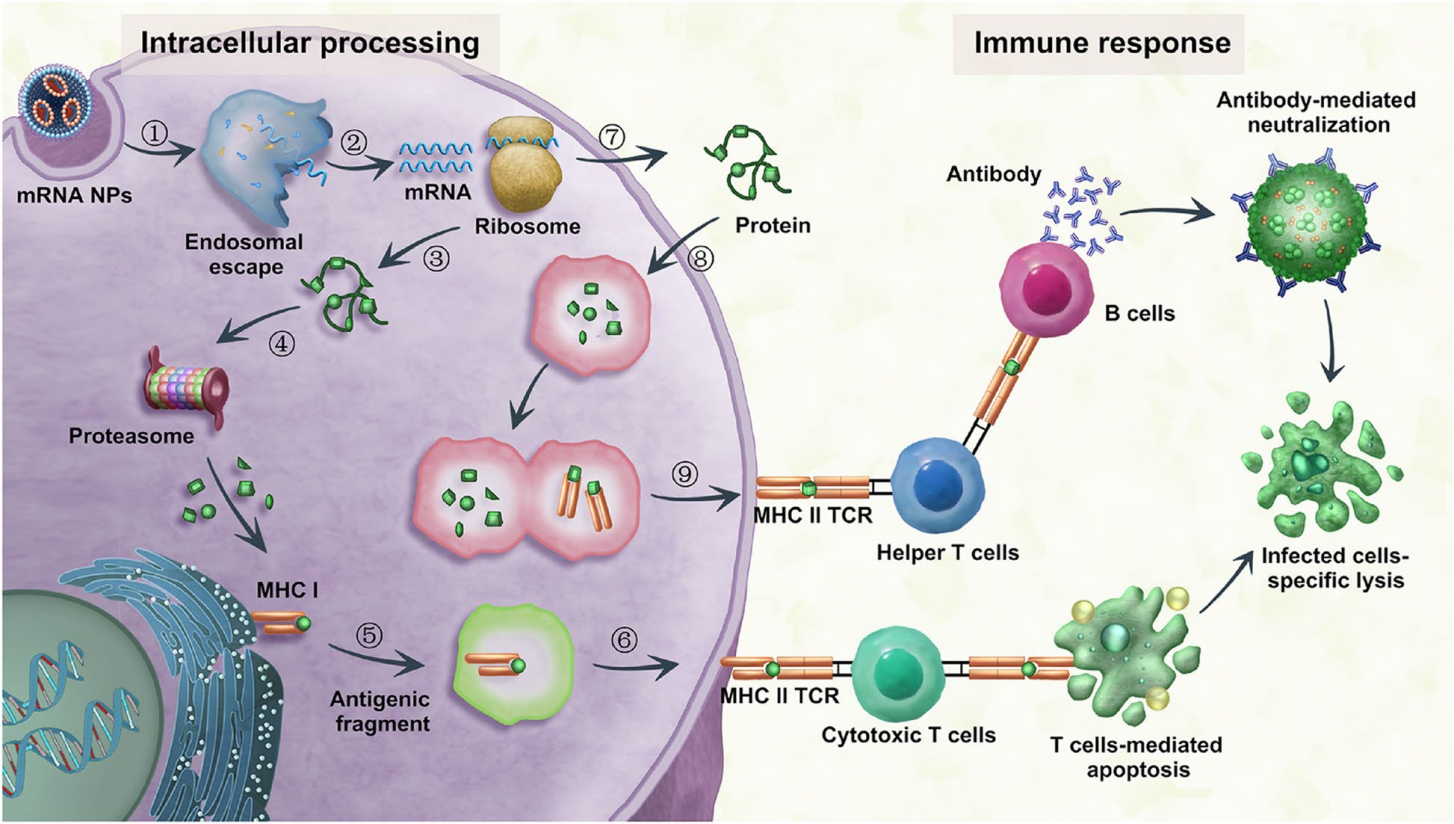
Mechanisms of immunity induced by mRNA NPs. Target cells endocytosis of mRNA NPs (①). Endosomal escape of mRNA into the cytoplasm (②). mRNA is translated into desired antigenic proteins by targeted cell ribosomes (③). Endogenous antigenic protein is degraded into polypeptides by a proteasome (④-⑤). Antigen peptides are presented to T cells in the form of antigen peptide-MHCImolecular complex and activate cellular immunity mediated by cytotoxic T cells (⑥). Besides, exogenous antigenic protein released earlier can be taken up by targeted cells and degraded (⑦-⑧). Exogenous antigenic protein can be presented by MHCIImolecular complex and activate helper T cells which stimulate B cell maturation to activate humoral immunity (⑨)

APCs, though few, are distributed throughout the body. Identification of pathogens by APCs is an important initial step to activate innate immunity and induce adaptive immunity (Fig. 13) [218]. After being taken up by APCs, mRNA NPs are translated into antigenic proteins in the cytoplasm and presented by the major histocompatibility complex (MHC) to corresponding T and B cells, inducing adaptive immunity. Their wide distribution throughout the body facilitates efficient regulation and activation of immune responses APCs directly targeting immune organs can enhance the efficiency and duration of immune responses [219].

The surface charge and size of LNPs have been found to affect their APC targeting. Sahin et al. [41, 220] designed an intravenously administered cationic liposomal RNA vaccine (RNA-LPX), composed of mRNA, the cationic synthetic lipid DOTMA, and the phospholipid DOPE. They found for the first time that RNA-LPX can be delivered to APCs in the lymph nodes, spleen, and bone marrow by adjusting the slight negative charge without having to modify ligands. It induced a robust immune response and showed promising therapeutic efficacy against melanoma [41], highlighting the potential of charge regulation as a simple yet effective strategy to enhance the performance of mRNA vaccines. Similarly, Nakamura et al. [221] reported that negatively charged LNPs, especially those with a small size (~ 30 nm), were favorable for efficient delivery to APCs in the lymph nodes after subcutaneous injection in the absence of nucleic acid cargoes. In contrast, the efficiency of LNPs larger than 100 nm for delivery to the lymph nodes was significantly reduced. They further suggested that PEGylation could prolong the circulation time of LNPs in vivo, increase their chances of being taken up by the lymphatic system, and promote the ability of LNPs larger than 100 nm to deliver to the lymph nodes [222]. Larger-sized LNPs (> 500 nm) could be taken up by DCs at the injection site, with the antigen delivered to T cells in the lymph nodes via the lymphatic route, which was typically slower and took longer than 24 h, so the LNPs could remain at the injection site [223].

**Fig. 14:**
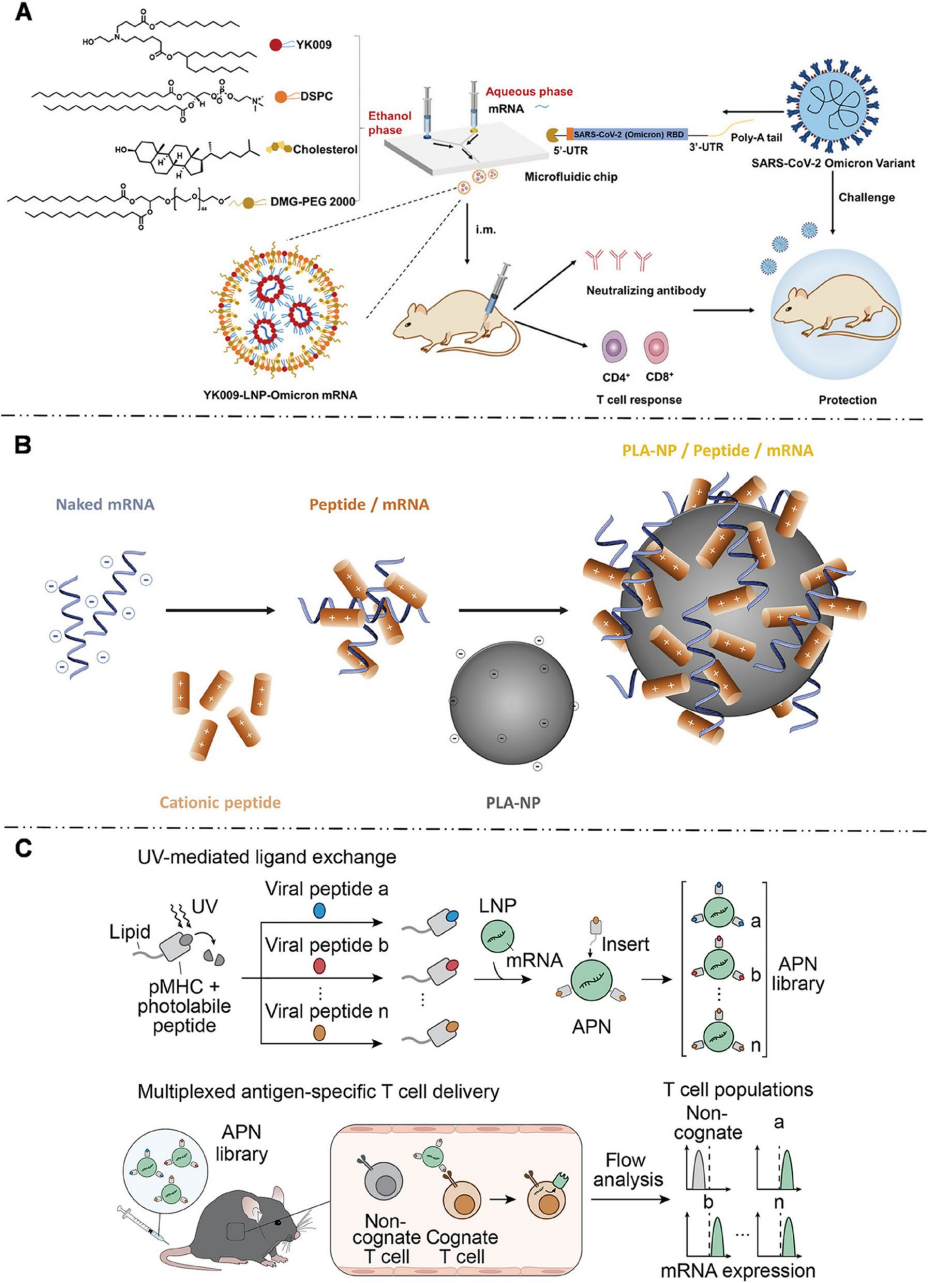
** A** Schematic illustration of effective humoral and cellular immune responses induced by YK009-LNP-Omicron mRNA vaccines to resist the Omicron variant of SARS-CoV-2. Reproduced with permission [208]. Copyright 2023, John Wiley and Sons. **B** Schematic illustration of PLA-NP/ cationic peptide/mRNA polyplexes. Reproduced with permission [224]. Copyright 2019, Elsevier. **C** Schematic illustration of UV-mediated peptide exchange of MHCI APNs for in vivo multiplexed delivery to virus-specific T cells. Reproduced with permission [217]. Copyright 2022, American Association for the Advancement of Science

mRNA delivery to DCs can also be enhanced without targeting ligands through optimization of ionizable lipids. Long et al. [208] developed a novel ionizable lipid YK009-LNP, whose ionizable part contains a tertiary amine headgroup, hydroxyl-modified at the end, with different ester bond positions and two different lengths of branched saturated tails, in the absence of targeting ligands. In vitro and in vivo studies showed that YK009-LNP-Omicron mRNA was internalized by DCs via CME and CvME, and mainly accumulated in the injection site and spleen of mice after intramuscular injection, inducing effective humoral and cellular immune responses (Fig. 14A). Furthermore, Ni et al. [209] found that piperazine-containing ionizable lipids (Pi-Lipids) could deliver mRNA to APCs, such as splenic macrophages, splenic DCs, and hepatic DCs, via intravenous injection. Chen et al. [49] designed a non-targeted LNP system 113-O12B that mainly delivered OVA mRNA to APCs in the lymph nodes after subcutaneous injection, inducing a robust antibody and CD8 + T cell response in a B16F10 melanoma mouse model and significantly inhibited the growth of OVA tumors in mice. Further, the tail length (≤ 12 carbons), linkage (ester bond), and amine head (including methyl) of active lipids enhanced the efficiency of mRNA delivery to the lymph nodes.

The efficiency of mRNA delivery and translation into APCs can also be improved by optimizing the composition of co-lipids and cholesterol. Medjmedj et al. [210] synthesized three groups of LNPs by varying the helper lipids (DSPC or DOPE) and cholesterol (Chol or β-sitosterol). The three groups were DSPC/Chol, DOPE/Chol, and DOPE/βS. DOPE/βS LNPs exhibited higher mRNA translation efficiency DCs than DSPC/Chol LNPs and DOPE/Chol LNPs in mouse.

Among active targeting strategies for LNPs, mannose is frequently employed as a ligand to design APC-targeting agents. Mannose-conjugated mRNA-loaded LNPs (mRNA-LNPs can selectively deliver mRNA to APCs, such as macrophages or DCs, and enhance immune responses regardless of the delivery route [225]. Besides mannose, antibodies or ligands against langerin, CLEC9A, and DEC205 have also been investigated as potential targeting agents for DCs and can be considered in the design of mRNA-LNP targeting [226–228]. However, the incorporation of targeting ligands complicates mRNA-LNPs formulation, causing significant challenges in clinical translation. Therefore, active APC targeting by mRNA-LNPs remains relatively limited.

Self-assembled cationic nano-micelles based on polyethyleneimine-stearic acid (PSA) copolymer and polycationic polyglucin-spermidine conjugates can both deliver encoded mRNA to DCs and induce neutralizing antibodies in mice [229, 230]. Recently, lipid-polymer nano-systems with lipid as shell and cationic polymer/mRNA as core were found to be highly efficient than single polymers in targeting DCs. With PLGA/mRNA as the core, the currently proposed lipid shells include DOTMA [231], DOTMA/DOPE [212], and PEG [232]. Lipid-PLGA/mRNA was able to transfect DCs and induce an immune response. Similarly, PbAE/mRNA polyplex core loaded in a lipid shell composed of multivalent cationic lipid (MLV5), DOPE, DSPE-PEG, and α-galactosylceramide (α-GalCer) improved mRNA delivery into DCs [233]. Under the combined action of antigen mRNA and immune adjuvant α-GalCer, it had an obvious therapeutic effect on the B16-F10 melanoma tumor. Further, Yang et al. [213] proposed a lipid-polymer NP system with core-shell structures encoding mRNA (LPP-mRNA) that is in phase I clinical trial in China (China Clinical Trial Registration Center, CTR20210542). The NPs localized the mRNA to the core with a cationic polymer named SW-01 and the outer shell coated with lipids. After intramuscular injection, the mRNA-NPs were highly selective to DCs and accumulated in the spleen and lymph nodes.

Additionally, certain peptides promote APC targeting. Intramuscular injection of cationic CPP-amphiphilic RALA motif peptide successfully transfected the antigen-encoded mRNA into DCs and induced antigen-specific T cell proliferation in vivo [65]. In a later study, Coolen et al. [224] reported that another complex composed of cationic cell-penetrating peptides (Lah4-L1) and PLA-NPs delivered mRNA to DCs in vitro better than did RALA-CPP (Fig. 14B). DCs absorbed them via phagocytosis and CME, regulated innate immune responses, and enhanced humoral and adaptive immune responses by activating endoplasmic and cytoplasmic pattern recognition receptors (PRRs). Fornaguera et al. reported a peptide-polymer mRNA-complex, which was composed of oligopeptide end-modified (OM)-PBAEs) [234]. After intravenous administration, OM-PBAEs specifically targeted and mainly transfected spleen APCs in vivo without aggregating in the liver.

##### T cells

T cells are generally the objects of antigen presentation by APCs, and it is difficult for T cells to take up mRNA NPs as easily as APCs. Therefore, targeting mRNA NPs to T cells is more challenging than to APCs. Most human T cells are located in lymphoid tissues, mucosal sites, and peripheral blood; i.e., mRNA via intravenous administration, rather than intramuscular administration, is required for sufficient T cell delivery [235]. Like extrahepatic targeting, direct targeting T cells must resist the liver-homing tendency of NPs. Due to the above obstacles, passive targeting of T cells is understudied. Patel et al. [211] reported that LNPs, replacing cholesterol with hydroxyl-containing cholesterol, can improve the mRNA delivery to T cells, which may be attributed to the alteration of the endocytosis circulation mechanism.

To precisely target T cells, active targeting is more suitable for mRNA delivery than passive targeting. T cells are divided into several subpopulations according to different CD molecules expressed on the cell surfaces. For example, helper T cells and regulatory T cells both express CD4, whereas cytotoxic T cells express CD8. CD3, CD5, or CD7 molecules on the surfaces. By attaching T cell–specific antibody fragments to the NP surface, some mRNA NPs have been developed for T cell targeted delivery. Parhiz et al. [192] reported that conjugating CD4 antibody to LNPs enabled specific targeting and mRNA interventions to CD4 + T cells. There are other similar strategies such as CD5-targeted LNPs and coupling an anti-CD8 antibody to polyglutamic acid/PBAE polymers [215, 216]. These mRNA NPs broadly target all T cells or T-cell subpopulations irrespective of antigen specificity, which contributes to a strong T cell response for applications in cancer therapy. Nevertheless, when facing certain viral infections or virus-mediated cancers, it is usually desirable to target only T cells specific to the disease instead of all T cells. Targeting only disease-specific T cells would help the immune system to maintain self-tolerance and avoid inflammation resulting from overactivity [235]. To accomplish this goal, Su et al. [217] developed an antigen-presenting NP (APN) for mRNA delivery through light-controlled synthesis. Different from the methods of antibody modification of LNPs surfaces, this kind of mRNA NPs exchanged MHC class I antigen peptides through ultraviolet (UV) light and bound with lipid tail specifically, to quickly generate different APN and transfected mRNA into homologous antigen-specific CD8 + T cells (Fig. 14C). This approach improves the precision with which mRNA-LNPs target and deliver mRNA to specific cytotoxic T cells.

### Others

#### Brain

A special structure located at the interface of blood and the brain is known as the blood-brain barrier (BBB) [236]. The BBB serves as a critical defense mechanism, shielding the central nervous system (CNS) from the invasion of macromolecules and other noxious substances. However, the BBB also poses a challenge to the delivery of mRNA NPs to the CNS. Current research efforts aim to overcome this hurdle, employing strategies such as direct injection into the brain parenchyma, intrathecal administration, nasal-to-brain pathway, and ultrasound-induced BBB opening. Lin et al. [237] were the first to suggest that n-Ethylpyrrolidone (NEP)-green fluorescent protein (GFP) mRNA loaded in a cationic polymer-based PEGylated nanocarrier could be delivered to the ventricle via intracerebroventricular (i.c.v.) administration, with protein translation achieved by diffusion. Peng et al. [238] utilized TransIT-mRNA, a commercially available transfection reagent composed of liposomes and polymers, to successfully deliver mRNA via intracranial injection and significantly inhibit the growth of glioblastoma. Nabhan et al. [239] achieved effective protein expression in the dorsal root ganglion via i.c.v. intrathecal delivery of mRNA-LNPs, proposing a potential treatment for Friedreich’s ataxia and other CNS diseases. PEG-associated toxicity and immunogenicity, potentially affecting mRNA delivery to the brain, have gained significant attention recently. To mitigate these issues, Bi et al. [240] optimized mRNA-lipopolymer formulations by replacing PEG with polysarcosine (pSar) and i.c.v. administering them to regulate intracerebral protein expression. The pSar- incorporating lipopolymers were found to avoid PEG- induced adverse immune reactions, while maintaining their stability and stealth properties.

Inhalation therapy through the nasal-to-brain pathway is an alternative mRNA delivery method to the CNS. Dhaliwal et al. [241] developed liposomes containing 1,2-dipalmitoyl-sn-glycero-3-phosphocholine (DPPC), DOPE, and cholesterol, and successfully delivered mRNA to the brain of CD-1 mice by the intranasal route. This approach improved the mRNA penetration rate and the accumulation in the cerebral cortex, midbrain cortex, and striatum.

Furthermore, some scholars have proposed techniques to induce transient BBB opening and deliver mRNA NPs to the brain, e.g., microbubble-assisted focused ultrasound (FUS) technology [242]. At an FUS irradiation intensity of 1.5 kW/cm^2^, the BBB was temporarily opened to deliver mRNA-LNPs to the brain via intravenous administration without side effects such as bleeding or edema. This provides a minimally invasive platform for brain-targeted mRNA delivery.

#### Bone tissue

**Fig. 15:**
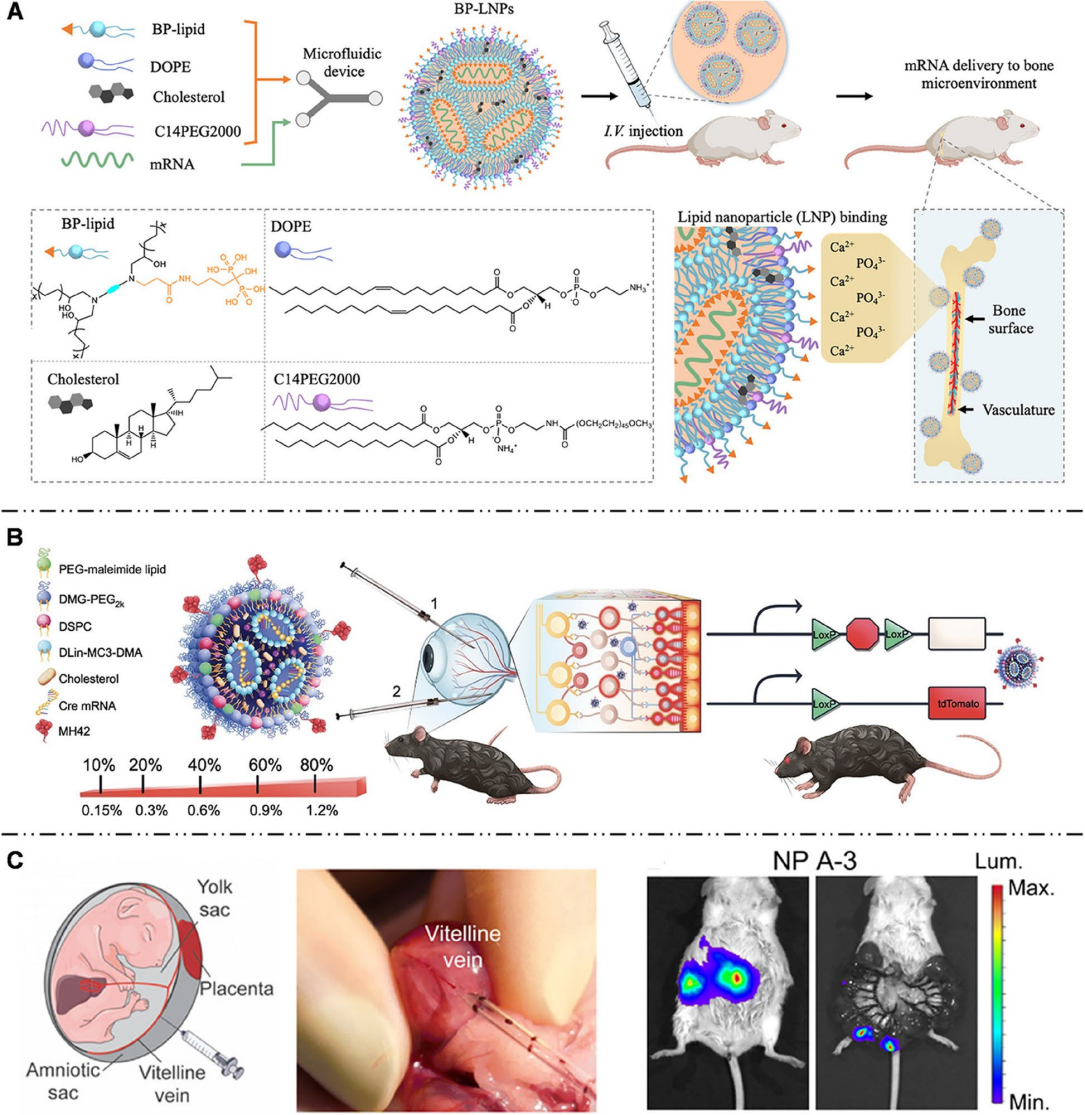
** A** Schematic illustration of the structure of BP-LNPs and the delivery of mRNA via intravenous injection to the bone microenvironment. Reproduced with permission [243]. Copyright 2022, American Chemical Society. **B** Schematic illustration of MH42 peptide–conjugated mRNA-LNPs based on intravitreal or subretinal administration. Reproduced with permission [247]. Copyright 2023, American Association for the Advancement of Science. **C** Practical operation of vitelline vein injection in mouse fetus and analysis of luciferase signal after fetal injection. Reproduced with permission [248]. Copyright 2021, American Association for the Advancement of Science

Bone is a dense tissue characterized by low blood flow and poor permeability. Currently, active targeting is the predominant strategy for delivering mRNA NPs to a bone via systemic administration. Bone tissues are primarily composed of hydroxyapatite (HA) and contain a small amount of organic collagen and water. Ligands that exhibit an affinity for HA, such as bisphosphonates (BPs), have been employed to synthesize BP-functionalized LNPs (BP-LNPs) (Fig. 15A) [243]. Upon intravenous administration, BP-LNPs deliver mRNA to the bone microenvironment in vivo, enhancing the secretion of therapeutic bone morphogenetic protein-2. Other bone-targeting ligands with high affinity for HA include tetracyclines (TCs) and oligopeptides [244, 245]. Additionally, collagen and various cell types present within the bone tissue and microenvironment, including bone marrow endothelial cells [246], osteoblasts, osteoclasts, osteocytes, stem cells, and immune cells, may serve as promising targets for mRNA NP delivery to the bone tissue.

Bones are present throughout the body, and bone-targeted NPs administered systemically may affect healthy bone tissues. Moreover, to achieve the desired concentration of NPs in the target site, the NP dose must be increased, which carries certain risks. In the case of bone defects, the process of bone tissue regeneration continues for weeks or even months. Therefore, it is crucial to ensure that mRNA NPs exhibit their pharmacodynamic efficacy specifically in the bone defect area, rather than other sound bone tissues. Some studies suggest that embedding mRNA NPs into biodegradable scaffolds and implanting them into bone tissue defects could be a solution. This approach prevents NPs from affecting healthy bone tissues in other parts of the body, while also ensuring control of dosage and mRNA NP release by adjusting the scaffolds’ degradation rate, which had successfully induced the osteogenic pathway of rat bone marrow stem cells [249].

Collagen scaffold, as a common carrier in bone defect, has been suggested for the sustained release of mRNA NPs, and guided the generation of new bone tissue for several weeks at the site of bone defects. For example, optimized mRNA encoding bone morphogenetic protein 2 (BMP2) was placed on the collagen sponge [250]; BMP2/ non-structural protein 1 (NS1) mRNA lipopolyplex was integrated into collagen-nano-hydroxyapatite scaffold, or CPPs-mRNA was uniformly distributed in a porous collagen scaffold [251, 252]. All these methods have effectively guided the formation of new bone at the site of bone defects. Despite these advances, bone-targeted mRNA NPs are still in the laboratory stage, and there remains a long way to go before their clinical application.

#### Eyes and heart

Systemic mRNA administration to the eyes has limited efficiency, making local administration a better option. LNPs are currently the primary nanocarriers for mRNA delivery to the eyes, and they can be administered locally through various routes, such as subretinal and intravitreal injections, as well as direct administration into the suprachoroidal space. The primary target cells are retinal pigment epithelial cells (RPE) and Müller cells, which have a significant impact on the pathological development of retinal diseases [253]. The peptide-guided LNPs are also proposed; they specifically deliver mRNA to photoreceptors in mouse models, offering a potential treatment option for inherited retinal degenerations (Fig. 15B) [247].

The delivery of mRNA-LNPs to the myocardium via intravenous injection has been achieved, however their level in the heart remains significantly lower than in the liver [254]. To enhance the targeting ability of systemic administration, identifying more effective myocardial targets or optimizing the carrier components is a feasible approach. Local injection (intramyocardial or intracoronary) may be a better option [255, 256], as it can minimize off-target effects. A new approach for heart-specific mRNA delivery is being developed to treat atrial fibrillation, and is currently in clinical phase I trial (NCT05223725). This study is evaluating the use of polymer hydrogels containing adenovirus-encapsulated mRNA, which are painted on the epicardium via surgery or interventional procedures. Although adenovirus is utilized as a vector in this study, it provides new insight on other nanocarriers as well. One advantage of this method is that mRNA NPs can exert efficacy continuously. Overall, the search for highly heart-specific mRNA NPs remains a focus in mRNA therapeutic research.

#### Fetus

Prenatal gene therapy, which includes protein replacement and gene editing therapies, is an emerging biotechnology. It holds promise for treating fetal congenital diseases in the pre-pathological or early stages, significantly reducing the incidence and mortality of these diseases [257]. Nonetheless, the implementation of a prenatal mRNA therapy targeting the fetus faces several limitations like those encountered in mRNA delivery to other organs, including mRNA instability and poor targeting ability. This hinders the clinical application of intrauterine mRNA therapy, highlighting the urgent need to explore novel mRNA delivery techniques.

The group of Michael J. has made great achievements in prenatal mRNA therapy by delivering ionizable LNPs (consisting of ionizable lipids, phospholipids, cholesterol, and lipid-anchored PEG) to the placenta or fetus in the uterus [248, 258, 259]. In one of their earliest studies, mRNA LNPs were administered to fetuses through the vitelline vein, and the organ distribution and mRNA delivery efficiency then was evaluated. The result showed that the mRNA was successfully delivered to the fetus’s liver, lungs, and intestines, thus providing a possible therapy for prenatal genetic diseases (Fig. 15C) [248]. This group also developed a novel strategy of amniotic fluid-stabilized LNPs, which could deliver mRNA into the fetus through intra-amniotic injection [258]. LNPs in the amniotic fluid, like those in the bloodstream, adsorb proteins, which has a potential impact on the targeting and stability of LNPs. Detailed proteomic analysis of various LNPs in amniotic fluid, based on the differences in the protein composition between amniotic fluid and blood, can improve the precise targeting of mRNA-LNPs.

Besides delivering mRNA into the fetus, Michael et al. also prepared vascular endothelial growth factor A (VEGF-A) mRNA LNPs for placental insufficiency. An LNP with ester bonds and negative zeta potential was synthesized. As mentioned earlier, in non-pregnant mice, ester bonds and negative charge promote mRNA-LNPs targeting the spleen. However, in pregnant mice, after intravenous injection of VEGF-A mRNA LNPs into the mother’s body, delivery of the VEGF-A mRNA shifted from the mother’s spleen to the placenta due to increased uterine blood flow and similar physiological features of the placenta and liver. The VEGF-A mRNA was then released to relieve placental ischemia [259].

In a recent study on prenatal mRNA delivery, Gao et al. [260] focused on the ability of LNPs to target extrahepatic organs. The results showed that at four weeks postnatally, 50.99 ± 5.05%, 36.62 ± 3.42%, and 23.7 ± 3.21% of myofiber in the diaphragm, heart, and skeletal muscles, respectively, were transfected, which displayed great potential for delivering mRNA to organs outside of the liver. Targeting the fetus’ heart is crucial as it is the target of multiple fetal diseases, and delivering mRNA to the heart is difficult to achieve in adulthood.

## mRNA NPs: administration routes

**Fig. 16:**
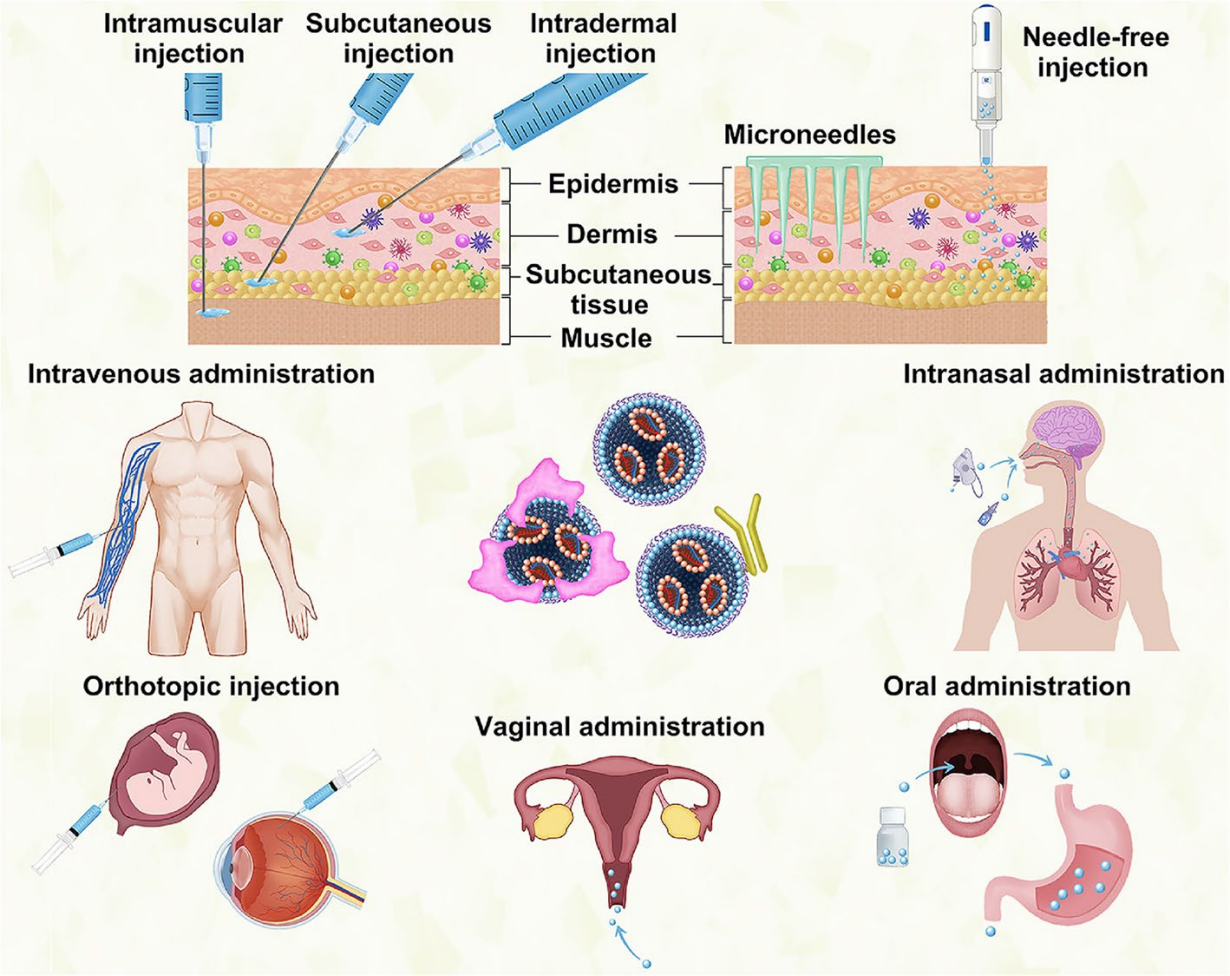
Schematic illustration of different administration routes for mRNA delivery

The administration route determines the barriers encountered by mRNA NPs before reaching the target organs, thereby influencing the biological distribution and targeting efficiency of NPs in vivo (Fig. 16). Notably, the severity of adverse effects with mRNA NP-based vaccines is also significantly influenced by the route of vaccine administration. As a result, the selection of administration routes is a primary consideration in the design of mRNA NP targeting strategies (Table 5).

**Table 5:** Summary of different types of administration routes for mRNA delivery

Administration Routes	Advantages	Disadvantages	Target organs/cells
**Intravenous administration**	Common methods of mRNA therapy Rapid onset of action High bioavailability mRNA NPs can be directly delivered to the systemic circulation	Invasive Extrahepatic targeting difficulty Immunogenicity	Liver Lung Spleen Lymph node Fetus
**Intramuscular (i.m.) administration**	Common method of vaccine administration Convenient Avoids first-pass metabolism	Invasive Painful	Immune cells
**Subcutaneous (s.c.) administration**	Convenient Avoids first-pass metabolism	Invasive Painful	Immune cells
**Intradermal (i.d.) administration**	Efficient delivery of small doses Minimal tissue damage	Invasive Painful	Immune cells
**Microneedles (MNs)**	Non-invasive Painless Simple operation Convenient storage Avoids first-pass metabolism	Specialized equipment required	Immune cells
**Needle-free injection (NFI)**	Non-invasive Painless Simple operation Avoids first-pass metabolism Contact areas between drugs and capillaries increased	Specialized equipment required	Immune cells
**Intranasal administration**	Aon-invasiveRapid onset of actionAvoids first-pass metabolism Precisely targeting the lungs Bypass the BBB and reach the central nervous system	Overcome barriers in respiratory tracts	Lung Brain
**Oral administration**	Non-invasive Convenient Easy to administer	Overcome barriers in gastrointestinal tract Low bioavailability first-pass metabolism	Gastrointestinal tract
**Vaginal nebulization**	Non-invasive Avoids first-pass metabolism Higher drug concentration with reduced dosage	Limited to female	Vagina Immune cells
**Intravesical instillation**	High local drug concentration Avoids systemic side effects Sustained release	Invasive Limited to bladder-related conditions	Bladder tissues
**Orthotopic injection**	Avoids some unique tissue barriers Precise delivery to target organ Avoids systemic side effects	Invasive Requires specialized expertise	Brain Eyes Fetus

### Intravenous (i.v.) administration

i.v. administration is a commonly employed approach for mRNA therapeutics due to its ability to deliver mRNA NPs directly into the systemic circulation, particularly for organs such as liver and spleen, which have a rich blood supply. Following their entry into the bloodstream, NPs encounter a complex environment.

Firstly, protein corona formation in the bloodstream leads to mRNA NP aggregation in the liver post i.v. administration, conferring natural advantages for liver-targeted mRNA NPs, which do not require the incorporation of active targeting ligands on their surfaces. However, when targeting non-liver organs in vivo, these mRNA NPs must resist this liver aggregation, which can be achieved through techniques such as adjusting the surface physicochemical properties of mRNA NPs, utilizing PEGylated NPs, and adding active targeting ligands.

Secondly, overcoming MPS clearance is crucial, as it is closely linked to the immunogenicity of mRNA NPs. In particular, NPs carrying mRNA encoding antigens, like mRNA vaccines for infectious diseases, can directly interact with non-specific immune cells in peripheral blood and be quickly eliminated. Thus, controlling the level of immune responses in peripheral blood is quite challenging [261].

Additionally, as mRNA vaccines circulate systemically throughout the body, they may cause undesired systemic adverse effects, rendering i.v. administration suboptimal for mRNA vaccination purposes.

### Local administration

#### Intramuscular (i.m.), subcutaneous (s.c.), and intradermal (i.d.) administration

The skin and muscle have abundant APCs and lymphocytes, making i.m., s.c., and i.d. administrations the most used methods for vaccination in clinical settings. Upon injection, immune cells are recruited to the injection site, where they take up NPs loaded with antigen-encoding mRNA. The antigens are presented to T helper cells and migrated to lymph nodes, which triggers an immune response. Short-term adverse effects, such as injection-site pain, systemic fever, and fatigue, are prevalent following mRNA COVID-19 vaccination via i.m. injection [262]. In the mouse model, changing the vaccination route from i.m. to s.c. was found to alleviate pro-inflammatory responses, while the humoral immune responses were unaffected [263]. Additionally, the cellular immune response with s.c. vaccination was superior to that of i.m. vaccination. Davies et al. [264] reported that incorporating a steroid prodrug into mRNA-loaded LNPs reduced the inflammatory response and significantly increased the level and duration of protein production after s.c. administration.

Studies have shown that i.d. mRNA vaccination is significantly more effective than i.m. administration. Specifically, one-fifth of the standard dose of i.d. booster could trigger T-cell responses comparable to the fractional i.m. boosters [265]. Moreover, i.d. administration leads to a more rapid antibody response than i.m. administration [266]. The strong immune response elicited by i.d. injection reduces the vaccine dose required and is a more cost-effective alternative to traditional i.m. injection. This effect may be attributed to the prolonged antigen retention and action time in vivo after i.d. injection [267]. Huang et al. [268] compared the efficacy of five different administration routes for the SARS-CoV-2 mRNA-liposomes (LPX/ receptor-binding domain [RBD]-mRNA) vaccine, including i.v., i.m., s.c., i.d., and intraperitoneal (i.p.) administration. They found that i.d. administration led to immunity earlier than i.v. and i.m. routes. Moreover, s.c., i.d., and i.p. injection routes were preferred for inducing Type 1 T helper (Th1) cells-biased immune responses. Each of the five administration routes induced immune responses at different speeds but elicited similar levels of antibody neutralization responses.

#### Microneedles (MNs)

Although needle injections are widely used for mRNA delivery, their side effects, such as infection and pain, can be unfriendly to patients, particularly those with needle phobia. Therefore, microneedle injection devices have been developed as a needle-free alternative for mRNA delivery [269].

Microneedles (MNs) are targeted DDSs that show the most potential for vaccine delivery currently [270]. Several tiny protrusions, composed of various materials including NPs, exist on their surface. They can painlessly penetrate the skin to deliver vaccines for treatment or prevention. MNs can achieve a robust immune response with painless vaccination. Moreover, MNs offer a safer alternative to needle injection, while also being convenient, offering high vaccine coverage, being patient-friendly and self-administered. Furthermore, MNs exhibit thermal stability, which provides a unique advantage for the development of COVID-19 vaccines [271]. This is particularly relevant given that most current COVID-19 vaccines require storage at low temperatures. Given the diverse range of materials available for MNs, biodegradable MNs have gained significant attention in the delivery of mRNA. They can degrade within the skin and release the drug over time without leaving sharp waste. Common biodegradable polymer components include polylactic acid, chitosan, and PLGA, which are also commonly used in mRNA delivery [272]. Recently, stimuli-responsive MNs, based on polymeric matrices, have also gained popularity in skin-targeted delivery research [273]. They can facilitate and control the release of payloads by internal and/or external stimuli [274]. For instance, changes in pH at the site of chronic wounds or cancer have been extensively studied as a stimulus for the design of pH-sensitive DDSs [275].

MNs based on mRNA are still in the research phase [272]. Despite the current lack of commercialized MNs, the consensus on MNs technology remains positive [276]. With in-depth research into mRNA NPs, the future of MN-based mRNA vaccines is bright.

#### Needle-free injection (NFI)

Like MNs, NFI is also a non-invasive injection method. NFI delivers medicines into the subcutaneous tissue by high-velocity jet [277]. The NFI system, which is a dispersed injection method, exhibited higher contact area than that of conventional needle administration between drugs and capillaries leading to more medicine absorption and better APC uptake [278]. NFI is extensively used in disease prevention and treatment, for instance, nucleic acid therapeutics and diabetes [279, 280]. Mao et al. [281] took inspiration from insulin injections in diabetics to introduce the NFI system (Quinovare, China) to the mRNA-LNP vaccines against SARS-CoV-2. The LNP was composed of ionizable lipid, DSPC, cholesterol, and PEG-lipid in optimized proportions. The NPs were surrounded by sucrose as the outermost layer, which made the conformation of NPs stable at high speed and pressure. The NFI-administered LNP-based mRNA vaccines had a diffuse tissue distribution, resulting in higher immunogenicity and effective cross-protection than i.m. administration. A clinical trial against rabies showed that participants vaccinated with prophylactic mRNA-based vaccines through a needle-free device exhibited higher antibody responses than those vaccinated through needle injection (ClinicalTrials.gov, number NCT02241135) [282].

### Intranasal administration

As one of the non-invasive ways, intranasal administration has unique advantages in the targeted treatment of respiratory and lung diseases with mRNA NPs. Being non-invasive, it avoids the side effects associated with acupuncture and significantly enhances patient compliance. There are also abundant immune cells near the nasal mucosa, which facilitates immunotherapy of mRNA vaccines. However, as mentioned in the previous chapter, mRNA NPs administered intranasally still need to overcome the barriers in the respiratory tract to reach the deep lung. Moreover, the latest research found that intranasal administration can bypass the BBB and reach the central nervous system through the sense of smell and trigeminal nerve, which provides a novel approach for brain or nerve treatment [283, 284].

### Oral administration

Oral administration of vaccines is a convenient and cost-effective way to deliver mRNA drugs. However, the protection of gastrointestinal mucosal barriers, digestive enzymes, and the extremely acidic pH level pose significant challenges that mRNA drugs must overcome to reach the target cells or organs. To address these issues, Abramson et al. developed a polymer carrier (PBAE) to encapsulate mRNA. These mRNA NPs were then delivered through orally administrated robotic pills [285, 286]. Following oral ingestion, the robotic pills could self-orient in the stomach, facilitating direct injection of mRNA NPs into the submucosa of the gastric mucosa to facilitate mRNA expression. Sung et al. [287] fed mice with the new LNPs (nLNPs) loaded with interleukin (IL)-22 mRNA in the mouse model of acute colitis, and observed an increase in the protein expression of IL-22 in the mice colon mucosa accelerating the healing process.

### Vaginal nebulization and intravesical instillation

Abundant immune cells are present in the vaginal mucosa. NPs can reach the proximal lymph nodes after vaginal administration in mice, which suggested that vaginal administration of NPs may be a potential method to deliver antigens to trigger immune response. When managing local inflammation, intravaginal administration of mRNA NPs can achieve higher drug concentration with reduced dosage than oral administration [288]. To overcome the vagina’s self-cleaning function, the delivery mode through the vagina generally chooses nebulization. In a study using pigs, intravaginal administration of mRNA NPs via nebulization was successful in protein translation, without causing apparent inflammation or irritation [289]. An important study of the mRNA delivery via vaginal nebulization was its use in inhibiting cervicovaginal human immunodeficiency virus (HIV) infections in sheep and rhesus macaques. These findings suggest that the application of mRNA delivery via vaginal nebulization has the potential for development in the treatment and prevention of infectious diseases [290].

The physical barriers of the bladder make it challenging to achieve targeted delivery of NPs via systemic administration. Nonetheless, intravesical instillation is a common administration route for bladder cancer therapy since it prevents unwanted protein circulation throughout the body and potential toxicity issues. Kong et al. [291] developed optimized mucoadhesive mRNA NPs that adhere effectively to bladder tissue via intravesical instillation. The optimized mucoadhesive mRNA NPs prolonged the exposure time of Lysine Demethylase 6 A (KDM6A)-mRNA in tumors and enhanced the penetration of NPs and KDM6A expression.

### Orthotopic injection

Tissue barriers in some unique organs, such as the BBB in the brain, the blood-ocular barrier in the eyes, and the fetoplacental barrier in the uterus, can impede the delivery of mRNA NPs to the target organs via intravenous injection. To circumvent this limitation, some studies opt to directly inject mRNA NPs into these solid organs. Direct injection sites in the eyes include the vitreous, suprachoroidal, and subretinal spaces. For the brain, mRNA NPs can be delivered through i.c.v. administration and intrathecal injection. Furthermore, mRNA-LNPs can be delivered via intra-amniotic injection and direct vitelline vein injection to the uterus of pregnant mice, resulting in protein translation in the fetal lungs, intestines, and liver [248, 258]. This provides a new delivery route for prenatal gene therapy.

## Conclusion and outlook

With the introduction of two COVID-19 vaccines, mRNA-based nanodelivery systems have achieved significant progress. Targeted mRNA NP delivery vehicles play a crucial role in the prevention and treatment of various diseases. The administration routes and the design of targeting strategies greatly influence the effectiveness of mRNA therapy. Precise targeted delivery to different organs and cells can be achieved by selecting appropriate administration routes, optimizing the physicochemical properties of mRNA NPs surfaces, and modifying ligands. This review presented common types of mRNA nanocarriers, targeting mechanisms, and cellular endocytosis pathways associated with precise delivery, design strategies for targeted mRNA NPs to different organs and cells, and different administration routes. Through rational design and development, innovative mRNA NPs hold promise for achieving remarkable clinical outcomes. Despite the tremendous potential demonstrated by targeted mRNA NP-based drug delivery, challenges persist. Here, we discussed the primary issues and developmental trends of targeted mRNA NPs.

### Administration routes

The administration route serves as the premise for the targeting design of mRNA NPs. Current mainstream administration methods include local and systemic routes. Local administration methods, such as subcutaneous and intramuscular injections, can reach high local mRNA drug concentrations, which have achieved some success in the vaccine field. Systemic administration, like intravenous injections, can deliver mRNA NPs throughout the body to treat multiple organs and lesions. For organs like the lungs, which possess both airway and vascular systems, the administration route determines the biological barriers faced by mRNA NPs, thus affecting the targeted delivery efficiency to different cells. Furthermore, certain unique barriers that cannot be traversed following systemic administration, such as the BBB, can be overcome by optimizing delivery routes. For instance, in situ injections to specific organs can deliver mRNA NPs to target organs and cells simply and effectively. The targeting capability can also be enhanced by introducing “third-party” approaches, such as microbubble-assisted FUS technology to open the BBB or using scaffolds at bone defect sites to release mRNA NPs. This area is worth further exploration. Moreover, non-invasive delivery methods, including intranasal administration, microneedles, needle-free injections, and vaginal nebulization, have increasingly attracted attention recently. These non-invasive delivery methods offer new insights into the targeting design of mRNA NPs.

### Extrahepatic delivery

The liver is the primary region where mRNA NPs tend to accumulate upon intravenous administration, which can be attributed to three main factors. First, the liver’s hemodynamics and unique sinusoidal fenestrated capillary structure increase the likelihood of mRNA NPs being captured by the liver. Second, the liver serves as the primary site for the metabolic clearance of mRNA nanoparticles, resulting in a significantly higher distribution of nanomaterials in the liver than in other organs. Lastly, due to the high expression of ApoE receptors in the liver, systemically administered mRNA NPs readily adsorb ApoE, leading to preferential liver accumulation. Therefore, the primary challenge in successfully delivering mRNA NPs to extrahepatic organs like spleen and lungs is overcoming the liver homing tendency of mRNA NPs.

Various approaches are employed to target mRNA NPs to extrahepatic organs, such as incorporating stealth components into mRNA NPs, with PEG being the most widely used stealth element to avoid liver uptake. Additionally, by modulating the physicochemical properties of mRNA NPs, such as size, pKa, charge, or adding active targeting ligands, different organs can be targeted. Moreover, for specific organs like the lungs or brain, selecting appropriate delivery routes can greatly enhance the targeting efficiency of mRNA NPs. Notably, some recent studies have achieved extrahepatic targeting by directly inducing the adsorption of other plasma proteins. Alternative stealth shells to PEG, such as poly(adenine), poly(2-oxazoline), and poly(amino acids), have been proposed to circumvent the side effects of PEG-specific antibody responses and PEG’s non-biodegradability. The extrahepatic targeted mRNA NP development provides valuable insights into overcoming liver homing tendencies, which contribute to design novel nanocarriers for other organs and cells.

### Cell-Specific targeted delivery

The targeting strategy of mRNA NPs gradually shifts from organ level to cell level, which is the future trend to achieve precise targeting. The study of endocytosis pathways and ligand-receptor-based active targeting can contribute to the design of cellular-level targeting strategies and improve the efficiency of targeted cellular uptake. According to the characteristics of target cells, appropriate endocytosis pathway can be selected to improve the uptake of mRNA NPs. The endocytosis pathway of cells can be regulated and guided by changing the protein corona adsorbed on mRNA NP surface to facilitate the targeted delivery of mRNA to different cells. However, this phenomenon remains unelucidated currently. Moreover, protein corona can shield targeting ligands, leading to targeting failure. This aspect should be carefully considered when designing NP targeting strategies. Future enhancement of the mRNA NP targeting ability may rely on a multimodal targeting approach combining passive, endogenous, and active targeting strategies.

### Targeting mechanism optimization

Optimization of mRNA NPs targeting strategies benefits from the continuous exploration and updates in targeting mechanisms. Passive targeting influences the fundamental design of organ-specific targeting strategies, requiring attention to the correlations between the physicochemical properties of mRNA NPs and the in vivo biological behavior or organ-specific barriers. Building on passive targeting designs, the primary research direction for active targeting is finding targeting ligands with high specificity, high affinity, and good biostability to minimize off-target effects and prevent the adsorption of protein coronas. For endogenous targeting, it is crucial to determine the relationships between protein corona composition, NPs properties, and organ or cellular targeting outcomes. With extensive research on protein corona profiling, recent efforts have focused on exploiting them rather than avoiding them. From current research, whether to utilize or avoid endogenous targeting based on protein coronas depends on the target organs or cells of mRNA NPs. However, there are still many challenges in the utilization of endogenous targeting. Firstly, there will be considerable differences in the content or types of protein coronas in vivo according to different diseases, which requires different disease models when studying protein coronas. Secondly, the differences of blood flow and protein composition between species need to be explored. This means that the information obtained in animal models may not be necessarily directly applied to humans, and it has been found that certain targeted delivery outcomes proven in small animal models may not be replicated in more advanced biological species. Due to species-dependent differences, the reliability of animal models in predicting endogenous targeting outcomes needs reconsideration. Research related to protein coronas in organ or cellular targeting is just beginning, and more studies are needed to elucidate these mechanisms to achieve better mRNA NPs targeted delivery.

### Influence of diseases and specific physiological conditions on targeting

Organ or cellular pathological conditions can impact the delivery efficiency of mRNA NPs. However, animal model studies based on disease states are still in their infancy, indicating the need for further investigation into the specific structures and physiological environments of organs under pathological conditions, as well as cell-specific receptors, to achieve the goals of precision medicine. Additionally, current mRNA therapies have proposed the capability to deliver multiple mRNAs within a single NP to achieve synergistic or complementary therapeutic effects for disease treatment. Nevertheless, this requires careful consideration of the potential trade-offs between effective payload capacity, NP size, stability, and targeted delivery efficiency.

Moreover, this article mentions a unique organ state, the uterus and fetus during pregnancy, which differs from normal organ conditions. Uterine blood supply significantly increases during pregnancy, and the placenta exhibits a porous endothelial structure like that of the liver. Additionally, the protein content in amniotic fluid markedly differs from that in serum. These factors greatly influence the delivery approach and design strategy for mRNA NPs targeting the uterus and fetus.

In summary, mRNA therapy represents a safer and more advanced approach than traditional therapies. Capitalizing on the benefits of NPs, they hold great potential as carriers for mRNA. Emphasis must be placed on the targeting capabilities of mRNA NPs to avoid potential off-target effects and associated side effects. Targeted therapies have shifted the design of mRNA delivery from a “one-size-fits-all” strategy to precision medicine.

## Data Availability

Not applicable.

## Abbreviations

ApoE: Apolipoprotein E
ASGPR: Asialoglycoprotein
BBB: Blood-Brain Barrier
CARTs: Charge-Altering Releasable Transporters
CF: Cystic Fibrosis
CME: Clathrin-mediated Endocytosis
CvME: Caveolae-mediated Endocytosis
DCs: Dendritic Cells
DDAB: Dimethyldidodecylammonium Bromide
DDS: Drug Delivery System
DODAP: 1,2-dioleoyl-3-dimethylammonium-propane
DOPC: 1, 2-dioleoyl-sn‑glycero-3-phosphocholine
DOPE: 1,2-dioleoyl-sn-glycero-3-phosphoethanolamine
DOTAP: 1,2-dioleoyl-3-trimethylammonium-propane
DOTMA: 1,2-di-O-octadecenyl-3-trimethylammonium-propane
DSPC: 1,2-distearoyl-sn-glycero-3-phosphocholine
FUS: Focused Ultrasound
GalNAc: N-acetylgalactosamine
HSCs: Hepatic Stellate Cells
i.d.: Intradermal
i.m.: Intramuscular
i.v.: Intravenous
LNP: Lipid Nanoparticle
LSECs: Sinusoidal Endothelial Cells
MC3: DLin-MC3-DMA
MNs: Microneedles
MHC: Major Histocompatibility Complex
MPS: Mononuclear Phagocytic System
MZ: Marginal Zone
NFI: Needle-Free Injection
NP: Nanoparticle
PACE: Poly(amine-co-ester)
PBAE: Poly(β-amino ester)
PECAM: Platelet Endothelial Cell Adhesion Molecule
PEG: Polyethylene Glycol
PEI: Polyethyleneimine
PLA: Poly(lactic acid)
PLGA: Poly(lactic-co-glycolic acid)
PS: Pulmonary Surfactant
RNase: Ribonuclease
RP: Red Pulp
s.c.: Subcutaneous
SORT: Selector ORgan Targeting
WP: White Pulp
